# Solids that are also liquids: elastic tensors of superionic materials

**DOI:** 10.1038/s41524-022-00948-8

**Published:** 2023-01-19

**Authors:** Giuliana Materzanini, Tommaso Chiarotti, Nicola Marzari

**Affiliations:** 1 Theory and Simulations of Materials (THEOS), École Polytechnique Fédérale de Lausanne, CH-1015 Lausanne, Switzerland; 2 National Centre for Computational Design and Discovery of Novel Materials (MARVEL), École Polytechnique Fédérale de Lausanne, CH-1015 Lausanne, Switzerland; 3 Present Address: Institute of Condensed Matter and Nanosciences, Université Catholique de Louvain, B-1348 Louvain-la-Neuve, Belgium

**Keywords:** Batteries, Atomistic models, Structure of solids and liquids

## Abstract

Superionics are fascinating materials displaying both solid- and liquid-like characteristics: as solids, they respond elastically to shear stress; as liquids, they display fast-ion diffusion at normal conditions. In addition to such scientific interest, superionics are technologically relevant for energy, electronics, and sensing applications. Characterizing and understanding their elastic properties is, e.g., urgently needed to address their feasibility as solid-state electrolytes in all-solid-state batteries. However, static approaches to elasticity assume well-defined reference positions around which atoms vibrate, in contrast with the quasi-liquid motion of the mobile ions in fast ionic conductors. Here, we derive the elastic tensors of superionics from ensemble fluctuations in the isobaric-isothermal ensemble, exploiting extensive Car-Parrinello simulations. We apply this approach to paradigmatic Li-ion conductors, and complement with a block analysis to compute statistical errors. Static approaches sampled over the trajectories often overestimate the response, highlighting the importance of a dynamical treatment in determining elastic tensors in superionics.

## Introduction

In the search for solid-state electrolytes (SSEs) that could replace the liquid organic electrolytes used today and improve safety in commercial Li-ion batteries^1^, a significant effort is being directed towards testing and improving ionic conductivity^2–5^, chemical and electrochemical stability^6,7^, and fast transport at the electrodes^8,9^. However, in order to address the manufacturing and the operando feasibility of all-solid-state batteries (ASSBs), a thorough understanding of the mechanical properties of the SSEs is also needed, and the mechanical stability of the electrolyte is a critical parameter for ASSBs^10–12^. First, the volume changes due to the storage of Li ions in the active materials can not be accommodated as in conventional liquid-electrolyte batteries, resulting in a considerable strain of the SSE and SSE/active-material composite during cycling^10,13,14^. This can easily turn out into mechanical degradation of the electrolyte, as observed for amorphous Li_2_S-P_2_S_5_ cycled with an Sn-anode^15^ or for *β*-Li_3_PS_4_ cycled with a Ni-rich NCM cathode^16^. Second, an SSE with high mechanical resistance would inhibit dendrite propagation and possibly enable the advent of ASSB technologies with superior energy density exploiting Li-metal anodes^17^.

The fracture toughness^18–22^, which quantifies the resilience of an SSE to be damaged under tensile stress, has been shown to be related to a high Young’s modulus^23,24^. On the other hand, the resistance of an SSE to dendrite growth has been related to a high shear modulus^25^, although quantification of this relationship is under debate^26^. Knowledge of the elastic behavior (bulk, shear, Young’s modulus, and Poisson’s ratio) of an SSE might thus be ground to predict its mechanical stability even outside the elastic regime. In this respect, softness has been historically regarded as a favorable property for the design of ASSBs technologies: soft materials, in particular sulfides, are easier to deform and thus expected to maintain good conformal contact with the electrodes^10,27–29^, allowing in addition for room-temperature pressure sintering^10^. However, a low stiffness of the SSE is not necessarily associated with good battery performance, as shown in the above-mentioned experiments on amorphous Li_2_S-P_2_S_5_ and *β*-Li_3_PS_4_^15,16^ and through fracture toughness measurements on amorphous Li_2_S-P_2_S_5_^23^. A theoretical continuum study based on non-linear kinematics models further shows that, perhaps counter-intuitively, compliant SSEs with Young’s moduli in the range of the sulfides (*E* = 15 GPa) are more prone to micro-cracking than typically brittle materials as oxide SSEs^24^. Elastic constants and conductivity are also closely related^30^, and enhanced vibrational^31,32^ and rotational^33–35^ degrees of freedom for the host lattice (typical of more compliant materials) have been shown to correlate with higher conductivities. Lattice-dynamics descriptors have been recently used to perform high-throughput screening of Li SSEs^36,37^, and conductivity has proven to increase in rotationally free SSEs, as is the case of the sulfide *β*-Na_3_PS_4_^38^ or some LISICON oxides^3^. The relation between lattice softness and conductivity is nevertheless not straightforward, since the first usually lowers both activation barriers and jump frequencies^11,39,40^, which in turn have opposite effects on the latter, giving rise to the Meyer-Neldel rule (also called compensation rule^39–42^). Speed-of-sound measurements in halide-doped argyrodites^39^ and Sn-substituted Li_10_GeP_2_S_12_^43^ show that, below a certain threshold of optimal lattice softness (or maximum conductivity), stiffer materials are better conductors^11^.

Routinely, first-principles calculations of elastic tensors and elastic moduli of SSEs exploit static finite-strain methods^28,44–48^, that fit the total energy^49–52^ or the stress^53,54^ with respect to strain, with energies and stresses obtained from DFT static calculations at different applied strains. Whereas these approaches are a powerful tool for the calculation of the elastic constants of ordered intermetallic alloys^49,55^, and obviously of single crystals with a defined structure^51,52^, they are in principle not appropriate for superionic materials, where the dynamical disorder plays a significant role and the standard picture of atoms vibrating around fixed equilibrium positions is not valid anymore^56^. In this respect, extracting the elastic tensor from the quasi-harmonic vibrational free energy^57^ under finite-strain deformations^58,59^ would not improve the picture, as it still assumes the existence of well-defined reference positions around which the atoms vibrate, which is clearly not the case for SSEs^56^. In a seminal paper, Parrinello and Rahman^60^ combined constant stress simulations^61,62^ with the thermodynamical fluctuation theory of the strain^63^ in order to estimate the elastic stiffness tensor from molecular dynamics. This method, which in the remainder of this paper will be referred to as the “strain-fluctuation method", builds on the knowledge of the dynamics and on the statistical convergence of the strain fluctuations over the simulation time^64^, and presents the important advantage of considering all the statistically relevant configurations of the atoms at a given temperature and pressure; it is therefore particularly appealing for superionic materials.

It is the purpose of this work to apply the strain-fluctuation method^60^ to the calculation of the elastic tensors and moduli of SSEs, choosing two benchmark systems, namely Li_10_GeP_2_S_12_^44,65,66^ and its oxide counterpart Li_10_GeP_2_O_12_^67–69^. For this, extensive and accurate first-principles molecular dynamics simulations are performed in the isothermal-isobaric ensemble at *T* = 600 K and *P* = 0, with a Nose-Hoover thermostat^70^ and a Parrinello-Rahman barostat^61,71^ using Car-Parrinello molecular dynamics^72^. The elastic tensors and moduli are extracted from the dynamical covariance of the strain over the trajectory, and a block analysis is provided to estimate statistical errors^73^. From the knowledge of the errors and the analysis of the elastic moduli convergence over the trajectories, we estimate the simulation length which is needed to produce reliable results. 0K and room-temperature moduli are extracted from *NPT* simulations at different temperatures using Wachtman’s law^74–76^. Finally, in order to compare the strain-fluctuation method with the static methods alluded to above, we provide additional static calculations, in which we distort fully relaxed snapshots sampled from the molecular dynamics trajectories fitting the Murnaghan equation of state (EOS) or the stress vs strain relation to obtain the elastic constants^51^.

The paper is organized as follows. In Section Results “Elastic tensors and moduli from the strain fluctuations" we discuss the isobaric-isothermal cell dynamics and we present the results for the elastic tensors and moduli of Li_10_GeP_2_S_12_ and Li_10_GeP_2_O_12_ obtained from the strain-fluctuation method. For comparison, in Section Results “Elastic tensors and moduli from static methods" we present the results for the elastic tensors and moduli obtained from static calculations, using fully relaxed snapshots from the molecular dynamics simulations. The main results of this paper are discussed and summarized in the Discussion.

## Results

### Elastic tensors and moduli from the strain fluctuations

We simulate two superionic materials, Li_10_GeP_2_S_12_ (LGPS) and Li_10_GeP_2_O_12_ (LGPO), each in two phases, namely the quasi-orthorhombic^69,77^ and tetragonal^65^ phases for LGPS (that we call o-LGPS and t-LGPS, respectively^69^), and the orthorhombic^67,68^ and tetragonal^69,78^ phases for LGPO (that we call o-LGPO and t-LGPO, respectively^69^). We use the supercells reported in refs. ^69^ and ^79^, having 100 and 50 atoms for the o- and t- structures, respectively (see Supplementary Figure 1 for a description of the structures). We recall here that, while t-LGPS, o-LGPS, and o-LGPO are existing compounds^65,68,77^, t-LGPO is a hypothetical one^69,78,80^, that we include in this work following a recent first-principles investigation highlighting its high Li-ion conductivity^69^. We use Car-Parrinello (CP) molecular dynamics^72^, based on Kohn-Sham density-functional theory (DFT)^81,82^ in the plane-wave pseudopotential formalism^83,84^, as implemented in the cp code of the QUANTUM ESPRESSO distribution^85^, in the isobaric-isothermal ensemble (*NPT*) with a Nose-Hoover thermostat^86^ and a Parrinello-Rahman barostat^61,62^. We use the Perdew-Burke-Ernzerhof (PBE) generalized-gradient approximation (GGA) functional^87^ and sample the Brillouin zone at the Γ point, as in refs. ^69,78^ (for an extensive review on the performance of PBE and other DFT functionals, see, e.g., ref. ^88^). The *NPT* CP method and the numerical details of these simulations are described in the Supplementary Methods. We test the reliability of the **k**-point sampling for the stress tensor, and show that the pressure and the off-diagonal elements of the stress tensor remain within ~10^−2^ GPa when calculated with an unshifted (2, 2, 2) **k**-point grid^89^ compared to Γ-only sampling (see Supplementary Tables 7 and 8). The above test also helps discussing possible finite-size effects in the MD simulations^90^. For these, we also study (at 600 K, with *NPT* CP dynamics, for ~70 ps) a 100-atom 2 × 1 × 1 supercell of t-LGPS, also sampled at Γ, and we extract the elastic moduli: comparison with the analogous results for the 1 × 1 × 1 cell shows that increasing the size of the simulation cell does not alter the elastic moduli significantly (see Supplementary Figure 10). In Supplementary Figures 3 and 4, we also report results for molecular dynamics runs with different barostat masses, showing that changing the barostat mass in a range of values around the standard theoretical suggestion^70,85^ does not change the cell fluctuations. The choice of the thermostat’s mass is already discussed in the Supplemental Material of ref. ^69^.

**Fig. 1:**
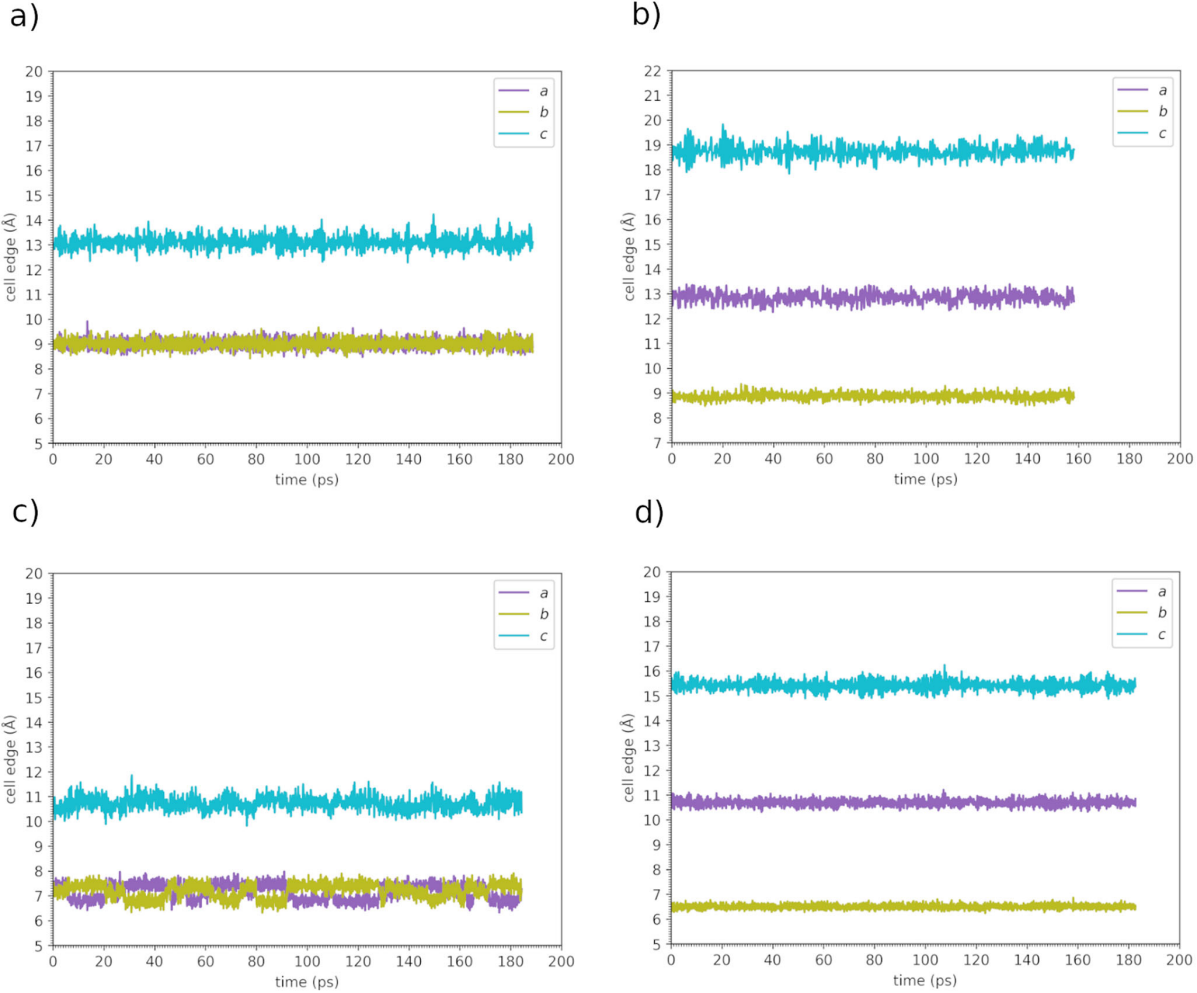
Cell edges (∥**a**∥, ∥**b**∥, and ∥**c**∥ in the text) in the 600 K-*NPT* CP molecular dynamics for the four structures studied. **a** t-LGPS; **b** o-LGPS; **c** t-LGPO; **d** o-LGPO. The cell angles and strain components are reported in Supplementary Figures 2 and 5, respectively.

In Fig. 1 we report the values of the cell edges ∥**a**∥, ∥**b**∥, and ∥**c**∥ of the four structures during a 600 K-*NPT* trajectory. While for t-LGPS, o-LGPS, and o-LGPO (Fig. 1a, b, d, respectively) these oscillate, for t-LGPO (Fig. 1c) ∥**a**∥ and ∥**b**∥ can swap during a fluctuation (as already reported in^69^). In Supplementary Figures 2 and 5 we also report the cell angles *α*, *β,* and *γ* from the same simulations, and the six components of the Voigt strain vector ***ϵ*** (see Equations (4)−(6) in the Methods Section), respectively. We recall here that **h** and ***ϵ*** in Equations (4)−(6) are time-dependent quantities. The diagonal elements of the strain-dynamical-covariance matrix (see also Equation (10) in Methods Section)

1 $$\left\langle {{\Delta }}{\epsilon }_{i}{{\Delta }}{\epsilon }_{j}\right\rangle =\left\langle {\epsilon }_{i}{\epsilon }_{j}\right\rangle -\left\langle {\epsilon }_{i}\right\rangle \left\langle {\epsilon }_{j}\right\rangle$$

are reported in Table 1. Larger fluctuations of the cell parameters translate into larger oscillations of the strain components, and in turn into larger values of $\left\langle {{\Delta }}{\epsilon }_{i}{{\Delta }}{\epsilon }_{i}\right\rangle$ (Table 1): an example is given by o-LGPS vs o-LGPO.

**Table 1:** Diagonal elements of the strain-dynamical-covariance matrix $\left\langle {{\Delta }}{\epsilon }_{i}{{\Delta }}{\epsilon }_{j}\right\rangle$.

	$\left\langle {{\Delta }}{\epsilon }_{1}^{2}\right\rangle$	$\left\langle {{\Delta }}{\epsilon }_{2}^{2}\right\rangle$	$\left\langle {{\Delta }}{\epsilon }_{3}^{2}\right\rangle$	$\left\langle {{\Delta }}{\epsilon }_{4}^{2}\right\rangle$	$\left\langle {{\Delta }}{\epsilon }_{5}^{2}\right\rangle$	$\left\langle {{\Delta }}{\epsilon }_{6}^{2}\right\rangle$
t-LGPS	3.90e-04	4.01e-04	3.81e-04	1.59e-03	1.61e-03	9.10e-04
o-LGPS	2.40e-04	1.88e-04	2.08e-04	3.94e-04	1.02e-03	5.18e-04
t-LGPO	1.89e-03	1.66e-03	6.13e-04	1.45e-03	1.42e-03	5.19e-04
o-LGPO	1.32e-04	1.38e-04	1.46e-04	2.25e-04	3.54e-04	2.70e-04

**Fig. 2:**
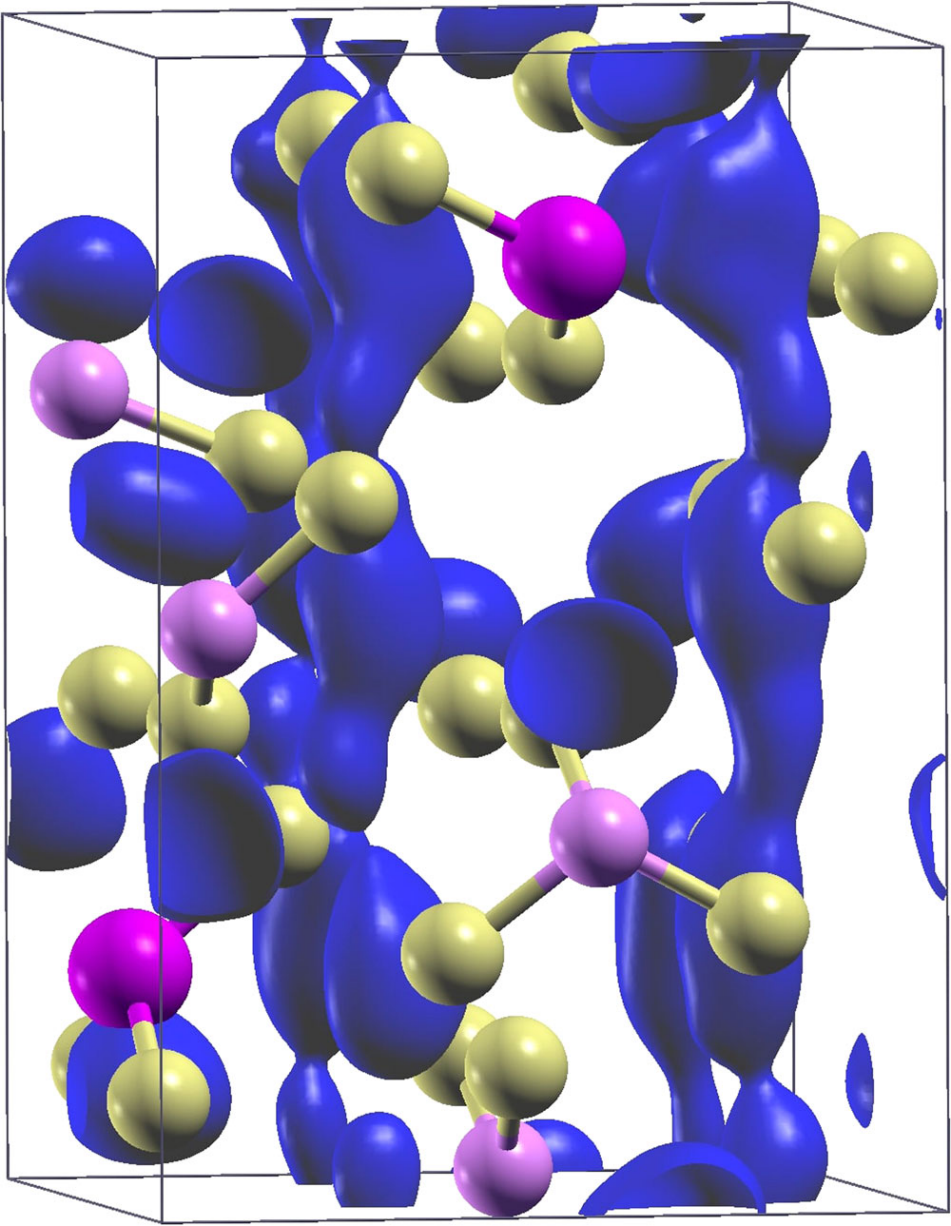
Li-ion probability density in t-LGPS from the 600 K-*NPT* CP molecular dynamics trajectory (0.08 Å^−3^ isovalues, blue isosurface). The equilibrium positions of sulfur, germanium, and phosphorus are shown as yellow, pink, and light rose spheres, respectively, and Ge−S and P−S bonds are displayed. Analogous Li-ion probability density isosurfaces are reported for o-LGPS, t-LGPO, and o-LGPO in Supplementary Figure 6.

To show the superionic character of these materials, in Fig. 2 an isosurface of Li-ion probability density (*ρ*(Li) = 8 × 10^−2^ Å^−3^) is displayed for t-LGPS from the 600 K-*NPT* CP dynamics (we use the implementation in https://github.com/lekah/samos, see also^91^). In Supplementary Figure 6, we report analogous isodensity plots for o-LGPS, t-LGPO, and o-LGPO.

**Fig. 3:**
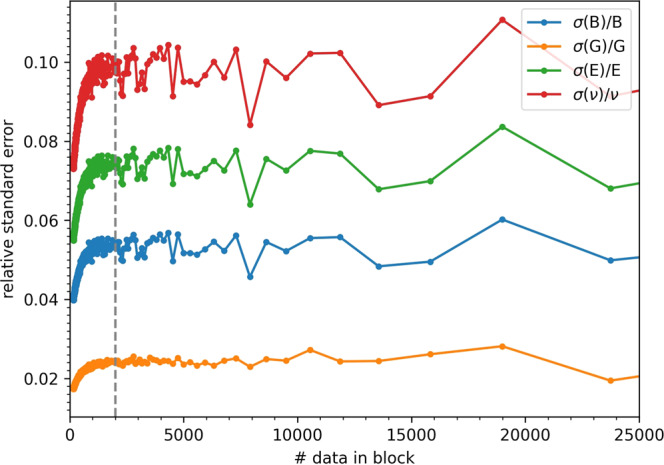
Relative standard deviation (square root of the variance of the elastic modulus divided by the value of the mean) of *B*, *G*, *E*, and *ν* as a function of the number of data in block used to calculate the variance of $\left\langle V\right\rangle$, $\left\langle {{{\boldsymbol{\epsilon }}}}\right\rangle$ and $\left\langle {{{\boldsymbol{\epsilon \epsilon }}}}\right\rangle$ (see Section Results “Elastic tensors and moduli from the strain fluctuations" and Section Methods), from the 600 K-*NPT* CP molecular dynamics of t-LGPS. Here, the first point on the right corresponds to four blocks, and the maximum number of blocks considered is 600. Based on this plot, we choose 47 blocks for the error block analysis of t-LGPS. Our choice is reported in the figure by the vertical dashed line (each block is ~4 ps long). Analogous plots for o-LGPS, t-LGPO, and o-LGPO are reported in Supplementary Figure 7.

**Fig. 4:**
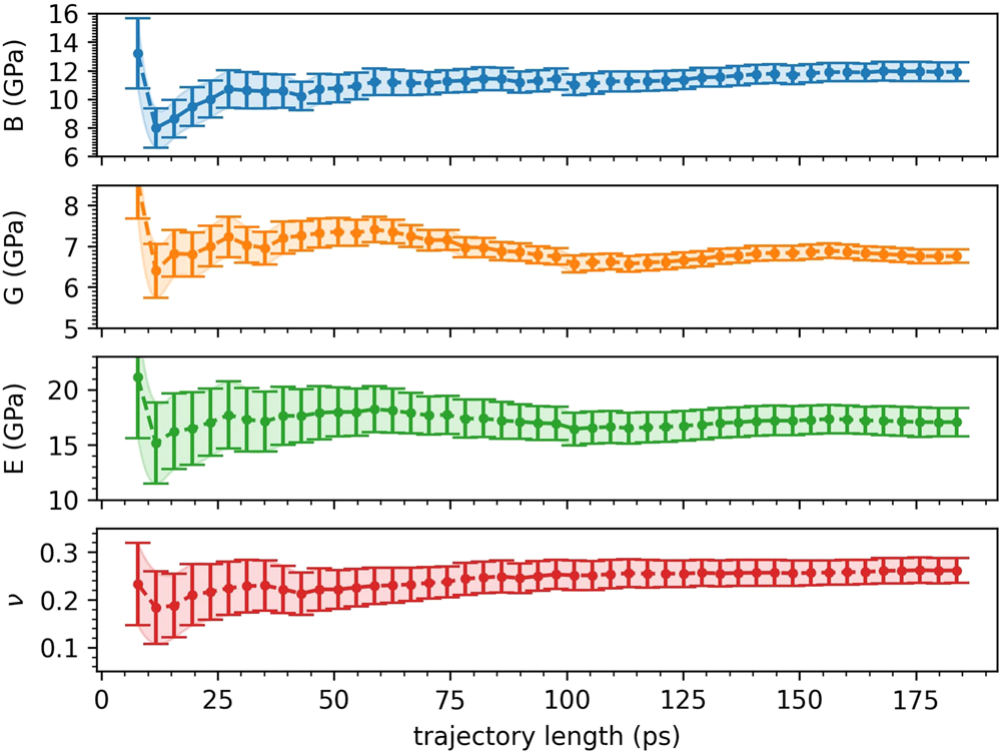
Dependence of the elastic moduli and Poisson’s ratio on the length of the 600 K-*NPT* CP molecular dynamics trajectory for t-LGPS. Each point corresponds to a trajectory which is *n*-block long, with *n* = 2, . . *N* blocks, *N* being the number of blocks that we have chosen for the whole trajectory, in this case 47 (see Fig. 3), each block containing the # data (~2000 data, ~4 ps) as determined from Fig. 3. The error bars are the standard errors of the moduli for each trajectory, obtained from the variance over the blocks, since these blocks are already uncorrelated and there is no need to repeat the block analysis in Fig. 3 for each of these calculations. Analogous plots for o-LGPS, t-LGPO, and o-LGPO are reported in Supplementary Figure 8.

The strain-dynamical-covariance matrix (Equation (1)) determines the stiffness and compliance elastic tensors from Equations (9) and (10) in the Methods Section, respectively. From the tensors, we obtain the moduli *B*, *G*, *E,* and *ν* following the Voigt-Reuss-Hill (VRH) approximation (see Methods Section). However, being the tensors statistical quantities, we need first to determine their statistical uncertainties over the trajectory length, and estimate sufficient trajectory lengths to give meaningful results from Equations (9) and (10). We compute the statistical errors on $\left\langle V\right\rangle$, $\left\langle {{{\boldsymbol{\epsilon }}}}\right\rangle$, and $\left\langle {{{\boldsymbol{\epsilon \epsilon }}}}\right\rangle$ in Equations (9) and (10) through a block analysis, i.e., by calculating the variance of the mean of a subset of data (blocks) of the whole set. A careful evaluation of the correct number of blocks is mandatory, so as to avoid error overestimation (too few uncorrelated data) or underestimation (correlated data)^73^. For each run, we set this number by performing a systematic partitioning of the trajectory in increasing number of blocks up to a maximum number, and by selecting the region where the variance of the mean is stable over the number of blocks^69,73^. This procedure is illustrated for the 600 K-*NPT* CP dynamics of t-LGPS in Fig. 3, where we report the relative standard error (square root of the variance of the elastic modulus divided by the value of the mean) for *B*, *G*, *E,* and *ν* (obtained from error propagation, see Methods Section) as a function of the number of data in block chosen for the block analysis of $\left\langle V\right\rangle$, $\left\langle {{{\boldsymbol{\epsilon }}}}\right\rangle$ and $\left\langle {{{\boldsymbol{\epsilon \epsilon }}}}\right\rangle$. By increasing the number of blocks (going right to left in the plot), the variance oscillates less strongly, and reaches a region of stability (here at ~ 2000 data in each block), after which it decreases monotonically (correlated data). This plateau determines the proper number of data in block^73^ and thus the error. Analogous plots for the evaluation of the number of blocks for o-LGPS, t-LGPO, and o-LGPO are reported in Supplementary Figure 7. In turn, from these trajectories, we identify 47 uncorrelated blocks for t-LGPS (each block ~4 ps), 41 uncorrelated blocks for o-LGPS (each block ~4 ps), 16 uncorrelated blocks for t-LGPO (each block ~10ps), and 31 uncorrelated blocks for o-LGPO (each block ~5 ps). Next, we study the convergence of the elastic moduli on the simulation time. For each system we perform several calculations of the elastic moduli, each of them using only a part of the whole trajectory simulated, corresponding to *n* = 2, . . *N* blocks, *N* being the number of blocks that we have chosen for the whole trajectory (see above), and each block having the same length as determined from the block analysis on the whole trajectory. Then, for each of these calculations we obtain the standard errors of the moduli from the variance over the blocks, since these blocks are already uncorrelated and there is no need to repeat a block analysis for each of these calculations. The values of the moduli as a function of the simulation time, together with the related standard errors are reported in Fig. 4 for t-LGPS, whereas for the remaining three structures they are reported in Supplementary Figure 8. In Fig. 4, the errors of *B*, *G*, *E,* and *ν* decrease, and their absolute values converge, while increasing the simulation time. A similar behavior is shown by o-LGPS and o-LGPO (Supplementary Figures 8a, c). For t-LGPO (Supplementary Figure 8b) we have a totally different scenario: the moduli are almost independent of the length of the trajectory, and their relative standard errors are very large, ranging from 10% (Poisson’s ratio) to 60% (Young modulus). In addition, the values of the moduli for t-LGPO are significantly lower than the respective moduli for o-LGPO (Supplementary Figures 8b and 8c). The behavior of t-LGPO is directly related to the cell dynamics of t-LGPO reported in Fig. 1, where ∥**a**∥ and ∥**b**∥ oscillate and swap: these fluctuations give rise to a material that would seem more compliant, but they would disappear if larger supercells were used. For this reason, we investigate a 4-times larger 2 x 2 x 1 supercell, already used in^69^, that we simulate for 100 ps: in Supplementary Figure 9 and Supplementary Table 6 we show that for this larger supercell the oscillations of ∥**a**∥ and ∥**b**∥ are considerably diminished, and the moduli higher. However, the statistical uncertainties remain high, and we conclude that for this material, where these two alternate phases can swap, even larger supercells should be used. For the remaining three structures (Fig. 4 and Supplementary Figures 8a, 8c), *B*, *G*, *E,* and *ν* can be considered reasonably converged from the reported simulations at 600 K for simulation lengths of the order of 150−200 ps.

The converged elastic tensors with errors, obtained from *NPT* (*T* = 600 K, *P* = 0) CP molecular dynamics through the analysis presented above, are reported in Table 2 for t-LGPS, o-LGPS, and o-LGPO. The space-group features of the tensors^92^ can be deduced: in particular, for both tetragonal and orthorhombic structures, the components in the off-diagonal blocks are ≃ 0, apart from *c*_15_ in the o-LGPO tensor, suggesting a certain monoclinic degree^92^ in this structure. For t-LGPS we observe *c*_44_ ≃ *c*_55_, *c*_11_ ≃ *c*_22_ and *c*_13_ ≃ *c*_23_. In Table 2 we also report the moduli (Voigt and Reuss bounds, and VRH average) with their statistical uncertainties. We note that the statistical uncertainties on the moduli are in general within ~2−8%.

**Table 2:** Elastic stiffness tensors and moduli (Voigt-Reuss bounds and VRH average) from the 600 K-*NPT* CP molecular dynamics and the strain-fluctuation method.

Material	Space group	Elastic tensor	$$\begin{array}{l}{B}_{{{{\rm{V}}}}}({{{\rm{GPa}}}})\\ {B}_{{{{\rm{R}}}}}({{{\rm{GPa}}}})\\ B({{{\rm{GPa}}}})\end{array}$$	$$\begin{array}{l}{G}_{{{{\rm{V}}}}}({{{\rm{GPa}}}})\\ {G}_{{{{\rm{R}}}}}({{{\rm{GPa}}}})\\ G({{{\rm{GPa}}}})\end{array}$$	$$\begin{array}{l}{E}_{{{{\rm{V}}}}}({{{\rm{GPa}}}})\\ {E}_{{{{\rm{R}}}}}({{{\rm{GPa}}}})\\ E({{{\rm{GPa}}}})\end{array}$$	$$\begin{array}{l}{\nu }_{{{{\rm{V}}}}}({{{\rm{GPa}}}})\\ {\nu }_{{{{\rm{R}}}}}({{{\rm{GPa}}}})\\ \nu ({{{\rm{GPa}}}})\end{array}$$
t-LGPS^65,78^	tetragonal (*P*4_2_*/nmc*)	$$\left(\begin{array}{cccccc}22.9\pm 1.8&7.7\pm 1.6&4.7\pm 1.3&-0.1\pm 0.5&-0.6\pm 0.6&-0.2\pm 0.7\\ 7.7\pm 1.6&23.3\pm 1.8&6.7\pm 1.6&0.4\pm 0.5&-0.3\pm 0.6&-0.2\pm 0.6\\ 4.7\pm 1.3&6.7\pm 1.6&23.0\pm 2.6&-0.2\pm 0.8&0.1\pm 0.8&-0.2\pm 0.6\\ -0.1\pm 0.5&0.4\pm 0.5&-0.2\pm 0.8&4.9\pm 0.4&-0.1\pm 0.2&0.3\pm 0.3\\ -0.6\pm 0.6&-0.3\pm 0.6&0.1\pm 0.8&-0.1\pm 0.2&4.9\pm 0.4&-0.2\pm 0.3\\ -0.2\pm 0.7&-0.2\pm 0.6&-0.2\pm 0.6&0.3\pm 0.3&-0.2\pm 0.3&8.6\pm 0.6\end{array}\right)$$	$$\begin{array}{l}11.9\pm 0.6\\ 11.9\pm 1.2\\ 11.9\pm 0.6\end{array}$$	$$\begin{array}{l}7.0\pm 0.3\\ 6.5\pm 0.2\\ 6.8\pm 0.2\end{array}$$	$$\begin{array}{l}17.6\pm 1.3\\ 16.5\pm 2.1\\ 17.0\pm 1.3\end{array}$$	$$\begin{array}{l}0.25\pm 0.02\\ 0.27\pm 0.05\\ 0.26\pm 0.03\end{array}$$
o-LGPS^67,69,77^	orthorhombic (*Pnma*)	$$\left(\begin{array}{cccccc}22.1\pm 1.5&13.0\pm 1.6&6.7\pm 1.3&0.1\pm 0.6&-0.1\pm 0.4&-0.4\pm 0.5\\ 13.0\pm 1.6&30.0\pm 2.2&9.9\pm 1.6&0.1\pm 0.7&-0.4\pm 0.5&-0.3\pm 0.6\\ 6.7\pm 1.3&9.9\pm 1.6&22.4\pm 1.6&-0.1\pm 0.5&0.7\pm 0.4&-0.7\pm 0.5\\ 0.1\pm 0.6&0.1\pm 0.7&-0.1\pm 0.5&9.9\pm 0.4&-0.1\pm 0.2&0.3\pm 0.3\\ -0.1\pm 0.4&-0.4\pm 0.5&0.7\pm 0.4&-0.1\pm 0.2&3.9\pm 0.2&0.2\pm 0.2\\ -0.4\pm 0.5&-0.3\pm 0.6&-0.7\pm 0.5&0.3\pm 0.3&0.2\pm 0.2&7.5\pm 0.3\end{array}\right)$$	$$\begin{array}{l}14.8\pm 1.0\\ 14.0\pm 2.2\\ 14.4\pm 1.2\end{array}$$	$$\begin{array}{l}7.3\pm 0.4\\ 6.4\pm 0.2\\ 6.8\pm 0.2\end{array}$$	$$\begin{array}{l}18.7\pm 2.0\\ 16.7\pm 3.5\\ 17.7\pm 2.1\end{array}$$	$$\begin{array}{l}0.29\pm 0.03\\ 0.30\pm 0.08\\ 0.29\pm 0.04\end{array}$$
o-LGPO^67,68^	orthorhombic (*Pnma*)	$$\left(\begin{array}{cccccc}77.2\pm 5.7&37.5\pm 5.0&20.9\pm 4.2&-0.6\pm 1.4&4.6\pm 1.7&0.8\pm 1.3\\ 37.5\pm 5.0&81.7\pm 5.6&31.3\pm 5.1&-0.4\pm 1.3&0.8\pm 1.7&-1.1\pm 1.2\\ 20.9\pm 4.2&31.3\pm 5.1&65.8\pm 7.1&-0.3\pm 0.8&-0.2\pm 1.9&-0.2\pm 0.9\\ -0.6\pm 1.4&-0.4\pm 1.3&-0.3\pm 0.8&34.4\pm 1.7&0.5\pm 0.5&0.9\pm 0.9\\ 4.6\pm 1.7&0.8\pm 1.7&-0.2\pm 1.9&0.5\pm 0.5&22.2\pm 1.4&0.9\pm 0.3\\ 0.8\pm 1.3&-1.1\pm 1.2&-0.2\pm 0.9&0.9\pm 0.9&0.9\pm 0.3&28.7\pm 0.9\end{array}\right)$$	$$\begin{array}{l}44.9\pm 2.1\\ 43.4\pm 4.8\\ 44.1\pm 2.6\end{array}$$	$$\begin{array}{l}26.1\pm 1.0\\ 24.7\pm 0.5\\ 25.4\pm 0.6\end{array}$$	$$\begin{array}{l}65.5\pm 4.8\\ 62.3\pm 9.1\\ 63.9\pm 5.2\end{array}$$	$$\begin{array}{l}0.26\pm 0.02\\ 0.26\pm 0.05\\ 0.26\pm 0.03\end{array}$$

**Fig. 5:**
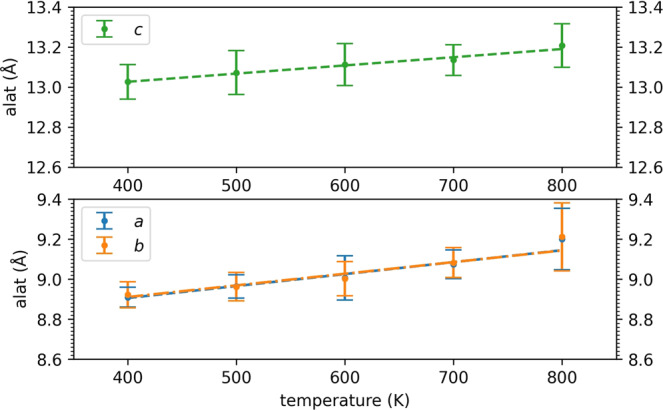
Temperature dependence of the t-LGPS lattice parameters (for o-LGPO see Supplementary Figure 11). The error bars are the standard deviations over the blocks, from a block analysis performed for each lattice parameter, as shown in Fig. 3 for the elastic moduli.

**Fig. 6:**
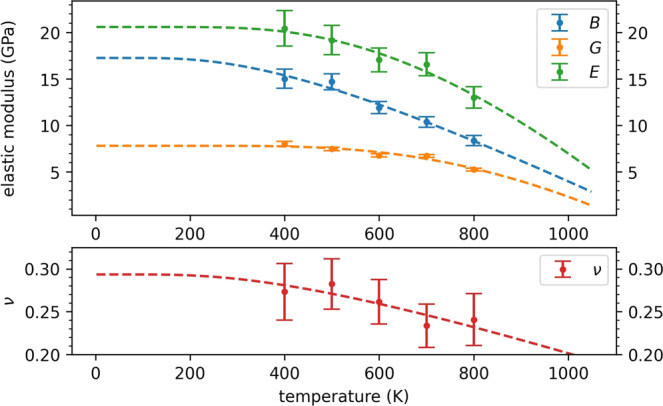
Temperature dependence of *B*, *G*, *E,* and *ν* for t-LGPS from *NPT* CP molecular dynamics at 400, 500, 600, 700, and 800 K. The error bars are the standard errors of the moduli over the trajectories, after a block analysis as from Fig. 3. The computed data are fitted to the Wachtman’s equation (Equation (2) and refs. ^74–76^). An analogous plot for o-LGPO is reported in Supplementary Figure 12.

Knowledge of the temperature dependence of the elastic moduli can provide room-temperature predictions to be compared with the experimental literature. To this end, we perform *NPT* CP molecular dynamics simulations at additional temperatures for t-LGPS and o-LGPO, from which we extract the elastic moduli, as described above for the simulations at 600 K. From these simulations we also extract the temperature dependence of the lattice parameters, that we report in Fig. 5, together with the linear fits, for t-LGPS (see Supplementary Figure 11 for o-LGPO). In Fig. 6, the values of *B*, *G*, *E,* and *ν* of t-LGPS as a function of temperature (*T* = 400, 500, 600, 700, and 800 K) are reported, together with a fit to the Wachtman’s law^74–76^:

2 $$M(T)={M}_{0}-\alpha T\exp \left(-\frac{{T}_{0}}{T}\right),$$

where $M=\left\{B,G,E,\nu \right\}$, *M*_0_ are the moduli at 0 K, and the meaning of the remaining parameters *α* and *T*_0_ is explained in^74–76^. This equation gives *M* = *M*_0_ at 0 K, approaching this value with a zero slope as required by the third law of thermodynamics^74–76^, and a linear dependence at high temperatures, as $\exp (-{T}_{0}/T)$ approaches unity. Although it was originally tested^74^ for some oxide compounds, it has been shown to be correct also for nonoxide solids^76^. An analogous plot for o-LGPO (*T* = 600, 800, 1000 K) is reported in Supplementary Figure 12. In Table 3 we report the extrapolated bulk, shear, Young’s moduli, and Poisson’s ratio at 0 K and 300 K for t-LGPS. For a non-quantitative reference, we also report the experimental room-temperature moduli of related sulfide glassy electrolytes, measured through ultrasonic pulse echo methods^93,94^. For o-LGPO, we report in Table 3 the values of the moduli at *T* = 300 K, that can serve as a reference for further studies, since, to date, no experimental reports on the elastic properties of this material are available.

**Table 3:** 0 K and room-temperature extrapolations, following a Wachtman’s fit, of bulk, shear, Young’s modulus, and Poisson’s ratio from *NPT* CP molecular dynamics and the strain-fluctuation method for t-LGPS and o-LGPO, together with some experimental results^93,94^.

	*B* (GPa)	*G* (GPa)	*E* (GPa)	*ν*	*B*/*G*
t-LGPS (0 K extrapolation)	17.25 ± 2.07	7.80 ± 0.66	20.58 ± 3.15	0.29 ± 0.05	2.21 ± 0.78
t-LGPS (300 K extrapolation)	16.46 ± 1.55	7.79 ± 0.59	20.48 ± 2.71	0.29 ± 0.04	2.11 ± 0.66
75Li_3_PS_4_ ⋅ 25Li_4_GeS_4_ (298 K^94^)	22.8	9.1	24.0	0.325	2.5
75Li_2_S ⋅ 25P_2_S_5_ (298 K^93^)	12.5−21.3	5.9−8.7	15−23	0.30−0.32	2.54−2.64
o-LGPO (300 K extrapolation)	46.74 ± 3.02	28.12 ± 0.87	69.59 ± 6.20)	0.26 ± 0.03	1.66 ± 0.40

### Elastic tensors and moduli from static methods

As a basis of comparison with the strain-fluctuation method, we provide here results from the static methods (outlined in the Methods Section) aiming to quantify the relevance of the dynamic nature of the elastic response of these materials. We focus on the benchmark material t-LGPS, for which results from computational static methods^28,44,45^ are also available in the literature, and a relevant number of experimental works investigate the mechanical properties of the class of superionics to which it belongs^23,26,93–96^. All the calculations are done both on nine uncorrelated snapshots of the 600 K-*NPT* CP molecular dynamics, that are previously fully relaxed, and on a global minimum energy structure that was obtained in ref. ^78^ through an electrostatic energy criterion, that is also previously fully relaxed. In order to compare the dynamics-based strain-fluctuation method with a static method at the same DFT accuracy, we perform here DFT calculations with the same supercell, pseudopotentials, DFT functional, cutoff energy, and **k**-point sampling as in the 600 K-*NPT* CP molecular dynamics simulations (see previous section, and the Supplementary Methods), and we relax the internal coordinates following a Broyden-Fletcher-Goldfarb-Shanno algorithm^85^, with, as convergence criteria, an energy difference between two consecutive steps below 2 × 10^−4^ a.u. and single components of the forces on the ions below 2 × 10^−3^ a.u.

**Fig. 7:**
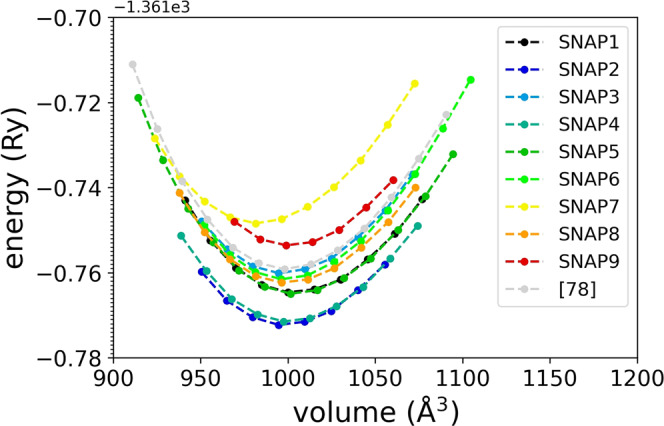
Energy-volume relations (and fit to the Murnaghan equation of state, Equation (15)) from isotropic compression and expansion of nine fully relaxed snapshots from the 600 K-*NPT* CP molecular dynamics of t-LGPS, and of the global minimum energy structure reported in ref. ^78^, previously fully relaxed. At each volume the energy is calculated by relaxing the atoms (see main text).

We apply a small hydrostatic strain (Equation (14) in the Methods Section) to each given structure of t-LGPS, calculate the energy, and fit to a Murnaghan EOS (Equation (15) in the Methods Section^85,97,98^), from which we extract *V*_0_, *B*_0_, and ${B}_{0}^{{\prime} }$. In Fig. 7 we report the energy-volume relations together with the fits, both for the nine snapshots and for the global minimum energy structure. In all these calculations, all the atoms are let free to relax during the expansion/compression, as explained above. However, in order to quantify the influence of the internal coordinate relaxation, we also perform the energy-volume calculations on the global minimum energy structure by keeping the atoms fixed at their equilibrium positions: we report the corresponding fit from these calculations, compared to the case with internal coordinate relaxation, in Supplementary Figure 13. The bulk modulus from the EOS is reported in the first line of Table 4 (B^*^(EOS)) for the unrelaxed calculations, and in the second line (*B*(EOS)) for the relaxed calculations. This comparison reveals that the material is about twice as stiff when the internal coordinates of the atoms are not free to relax. As for the energy-volume curves and bulk moduli obtained from the snapshots configurations (Fig. 7 and Table 4), the nine uncorrelated snapshots from the t-LGPS dynamics reveal a rather wide range of values (~15% of the average value) for the t-LGPS bulk modulus from the EOS, corresponding to rather different energy-volume curves. This result is not unexpected, as superionic materials can have different stable structures, mainly depending on which sites are populated by the mobile Li ions in each of them. Since there are 32 sites for 20 Li ions in one 50-atom supercell^99^, the number of such structures is huge (~10^8^), and it would not be possible to assign a proper statistical uncertainty to the moduli from the static methods in Table 4 from a block analysis, as done for the strain-fluctuation method in the previous section. However, from the block analysis of the previous section, we know that, by considering 9 uncorrelated snapshots (i.e., separated at least by the length of one block, which is ~4 ps from Fig. 3), we are not underestimating the statistical uncertainty of the static moduli in Table 4. Thus, sampling of the snapshots from the 600 K-*NPT* CP molecular dynamics is performed here as a way to generate uncorrelated t-LGPS configurations, and not as a rigorous statistical tool, which would be inconsistent as the energy-volume calculations are at 0 *K*. Finally, we note that the bulk modulus for the global minimum energy structure^78^ (last column in Table 4) is very similar to the average value obtained from the fully relaxed snapshots.

**Table 4:** *B*, *G*, *E* (in GPa), and *ν* from the static methods (EOS and stress–strain) applied to nine uncorrelated snapshots, fully relaxed, from the t-LGPS *NPT*-600 K CP molecular dynamics (with the standard errors of the mean), and to the global minimum energy structure^78^, fully relaxed.

	SNAP1	SNAP2	SNAP3	SNAP4	SNAP5	SNAP6	SNAP7	SNAP8	SNAP9	SNAP_AVE_	0 *K* global minimum^78^
B^*^(EOS)											48.0
*B* (EOS)	20.9	21.4	21.6	20.6	20.6	23.3	21.1	21.0	22.7	21.5 ± 0.3	21.7
*B* (stress–strain)	21.2	20.4	21.8	20.5	21.0	21.7	21.8	22.3	23.5	21.5 ± 0.3	21.7
*G* (stress–strain)	11.3	12.7	13.2	12.5	11.2	13.0	13.0	12.2	11.7	12.1 ± 0.3	13.3
*E* (stress–strain)	28.8	31.6	32.9	31.1	28.6	32.4	32.6	30.9	30.0	30.6 ± 0.6	33.0
*ν* (stress–strain)	0.27	0.24	0.25	0.25	0.27	0.25	0.25	0.27	0.29	0.26 ± 0.01	0.25

**Fig. 8:**
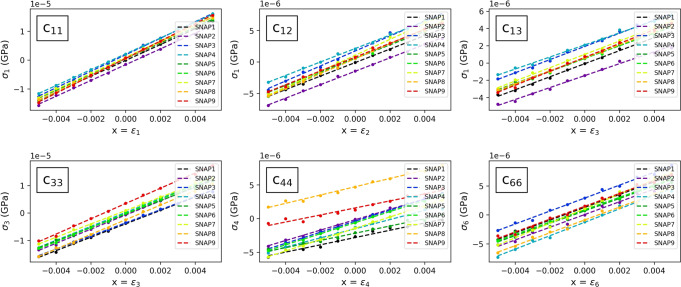
Stress–strain relations (Equations (18) and (19)), with linear fits providing the components *c*_11_, *c*_12_, *c*_13_, *c*_33_, *c*_44_, and *c*_66_ of the elastic tensor from Equation (19), for the same nine fully relaxed snapshots from the 600 K-*NPT* CP molecular dynamics of t-LGPS as in Fig. 7. At each value of the strain parameter *x* the stress is calculated by relaxing the atoms. The full elastic tensors for the nine configurations are reported in Supplementary Tables 2–4.

In order to calculate also *G*, *E*, and *ν*, we apply uniaxial strains to extract the 36 stiffness coefficients of the elastic tensor (Equations (18) and (19) in the Methods Section). For these calculations, we tighten the convergence criteria to energy differences between two consecutive steps below 2 × 10^−5^ a.u. and single components of the forces on the ions below 2 × 10^−4^ a.u. In Fig. 8 we report the components *c*_11_, *c*_12_, *c*_13_, *c*_33_, *c*_44_, and *c*_66_ of the stiffness tensor for the nine fully relaxed snapshots considered. The resulting moduli from Equations (20)−(22) are reported in Table 4, together with the moduli obtained from the same stress–strain calculations on the global minimum energy structure of ref. ^78^, here fully relaxed. In Supplementary Tables 2–5 we report the full elastic tensor and Voigt-Reuss bounds for each relaxed snapshot configuration, and for the global minimum energy structure. For the snapshot configurations, similar considerations as for the bulk modulus from EOS can be drawn for the moduli from the stress–strain relations: we find values of each elastic modulus distributed over a range of about 15% of the average value. As noted for the calculation of the bulk modulus from the EOS, the moduli obtained from the global minimum energy structure^78^ (last column in Table 4) do not give additional relevant information, being in the range of the moduli from the fully relaxed snapshots. This result can be understood as a demonstration that the global minimum energy structure and the energy-minimized snapshots are simply different energy-minimized configurations, each with a potentially different Li occupation of the available sites.

## Discussion

This paper provides reference results for the elastic constants and moduli of two benchmark oxide and sulfide SSEs from the strain-fluctuation method^60^. From Tables 2 and 3, the oxide o-LGPO^67,68,100^ (whose elastic properties were experimentally and computationally unreported, to date) is predicted to be significantly stiff (*B* = 44.1 GPa, *G* = 25.4 GPa, *E* = 63.9 GPa), and ~3 times stiffer than the corresponding sulfide. This result is compatible with the available results for the garnet Li_7_La_3_Zr_2_O_12_ (*B* ~ 100 GPa, *G* ~ 60 GPa, *E* ~ 150 GPa^47^) and the NASICON Li_1.2_Zr_1.9_Sr_0.1_(PO_4_)_3_ (*E* ~ 40 GPa^101^), showing a superior stiffness of oxide SSEs as compared to sulfide SSEs (see Table 3 for Li_3_PS_4_^93^ and LGPS^94^). However, these results do not univocally determine the relative performances of oxides and sulfides in ASSBs. Even though a large shear modulus has been historically believed to prevent Li penetration through the electrolyte^25,102^, a SSE that possesses a high *G* of ~60 GPa, LLZO, has recently proven to suffer from dendrite propagation^26^. Similarly, despite a low Young’s modulus is usually believed to ensure stress–strain-accommodation ability^27,102^, a compliant SSE with a low *E* of ~18 GPa, Li_3_PS_4_, has recently appeared to be more prone to micro-cracking than stiffer electrolytes^23,24^. A major goal for the scientific community would be to determine which balance of low Young’s modulus and high shear modulus leads to the best performance in reducing dendritic growth and interface resistivity. To this end, our results for o-LGPO seem to show a common trend with other oxide superionics, such as LLZO^47^ and Li_2_O-ZnO-B_2_O glasses^103^, where *E* ~ 1.5*B* and *B* ~ 1.5*G* (Table 3), differently from sulfide materials, for which this trend is not reported (this work and refs. ^28,93,94^), and in principle *G* and *E* might be more easily tuned to reach such ideal balance. Moreover, from the moduli, we can estimate the ductility, usually related to the ratio *B*/*G*^104^ (also called the Pugh’s ratio^105^), which quantifies the ability of a material to resist volume changes against shape changes. The *B*/*G* ratio for o-LGPO (≃1.7, Table 3) is in line with the one of another oxide SSE, the garnet LLZO^47^. The ductility of t- and o-LGPS is considerably higher (>2, Table 3), in line with the above-mentioned experiments for glassy solid-state sulfides^93,94,102^. A close comparison with the experimental literature (refs. ^27,93,94,102^ and Table 3) shows a good agreement with ref. ^94^ for the values of *G* and *E* and a fair agreement for the values of *B* and *ν* (Table 3). However, we recall that the experimental values of ref. ^94^, also reported in Table 3, refer to the elastic moduli of glassy Li_2_S − P_2_S_5_ and Li_3_PS_4_ − Li_4_GeS_4_^27,93,94^, and no experimental investigations of the elastic properties of single-crystal or polycrystalline LGPS are available to date. In addition, ref. ^93^ shows that the experimental moduli are very sensitive to the molding condition, being higher for higher molding temperature and pressure, and covering a wide range of values (e.g., for the composition 75−30 of Li_2_S − P_2_S_5_, see Table 3, *B* = 12.5−21.3 GPa, *G* = 5.9−8.7 GPa, and *E* = 15−23 GPa^93^). For the glassy Li_3_PS_4_ − Li_4_GeS_4_, since the hot-pressed pellets showed higher Li density and ionic conductivity^106^, the elastic moduli were measured only after hot pressing^94^, so that ref. ^94^ (cf. Table 3) reports only the upper limit of the experimental moduli for these glassy LGPS samples. Finally, it would be interesting to clarify the connections between hot- and cold-pressed glass structures, pure crystals, and elastic moduli.

A more theoretical purpose of this work is to compare the strain-fluctuation method exploiting *NPT* molecular dynamics trajectories (Tables 2 and 3) with static methods applied to fully relaxed snapshots extracted from the same trajectories. We purposely use here the same DFT machinery (DFT functional, pseudopotentials) and choose the same accuracy (**k**-point sampling, supercell size) in both methods. Moreover, we do not aim here to establish the accuracy of the DFT functionals^45,88^, but rather to compare the accuracy of static and dynamic methods. Such a comparison, that we perform for t-LGPS (Table 4), is expected to shed light on the role of the many statistically accessible configurations in the determination of the elasticity in superionic materials, and to clarify the need for a dynamical treatment in the description of their elastic response. First, Table 4 shows that the static methods give rather different values for the moduli, depending on the choice of the configuration to be strained. This result confirms that the different stable structures of superionic materials, which are dependent on the sites populated by the mobile Li ions, give rise to statistically different elastic properties, and is in principle not correct to use as a reference only one of these structures^44,45,107^. Furthermore, we find a marked disagreement between the average moduli from the static calculations (Table 4) and the 0 K extrapolation of the moduli from the strain fluctuations (Table 3), in general, the first exceeding the latter by a considerable amount. The static calculations performed on the global minimum structure^78^ are in line with those on the fully relaxed snapshots. The overestimation of the moduli from the static methods with respect to the strain-fluctuation method can be explained by the ability of the latter to capture the elastic response of superionic materials, where the non-mobile sublattice responds elastically as in a proper solid, whereas the mobile sublattice behaves inelastically as in a liquid. Conversely, the static methods assume the presence of an elastic response from all the atoms in the material, predicting in turn a stiffer material than it is in reality. For t-LGPS, our results show that such overestimation amounts to ~25–50%, so a proper statistical dynamic treatment of the elastic properties of this material should be desirable. Overall, we show that the elastic response of the material is systematically stiffer when going from a dynamic method (strain-fluctuation), to a static method (EOS or stress–strain) performed by relaxing the internal coordinates, to a static method (EOS) performed at fixed internal coordinates. Thus, relaxing the internal coordinates when performing a static calculation for the elastic moduli is beneficial for these materials (we show that otherwise the bulk modulus would be ~100% stiffer), but is not enough when compared to the correct dynamical treatment (since the moduli are still ~25–50% stiffer than the dynamically obtained ones).

In summary, although superionic conductors are dynamically disordered materials, for which a well-defined microscopic reference configuration does not exist^56^, computational studies of their elastic properties usually rely on static methods^28,44–48^, where strains are applied to a chosen ionic configuration (in general, one of the many possible fully relaxed structures starting from molecular dynamics trajectories or educated guesses from the experimental structures), and the resulting energies or stresses are calculated. Such methods neglect the quasi-liquid motion of these materials, which can instead be captured by turning to statistical methods, based on the sampling of the whole configuration space with molecular dynamics. First, we provide a computational study of the elastic moduli of superionic conductors with such a dynamical approach, sampling strain fluctuations^60^ with first-principles molecular dynamics^71,85^, followed by an accurate block analysis of the errors. Choosing two benchmark crystalline superionic conductors, LGPS^65^ and LGPO^68^, we show that an affordable computational effort is sufficient (~180 ps trajectories) to obtain converged moduli and statistical errors of reasonable size; the calculated moduli agree with the existing experimental literature for similar glassy materials. Second, we compare the strain-fluctuation method with standard static methods for the benchmark t-LGPS. By applying the same static methods to different fully relaxed structures from the molecular dynamics, both with and without internal coordinates relaxation, we note that: (i) static methods predict a material unrealistically stiff when unrelaxing the internal coordinates; (ii) static methods still overestimate the moduli with respect to the correct dynamical treatment by ~25–50% when relaxing the internal coordinates; (iii) static methods have an intrinsic variance up to ~2–3%, and an overall spread of ~15% on the average value. These results argue for the importance of dynamical sampling to address elastic properties of superionic conductors, and provide a computational reference for the community, given that no experimental reports on crystalline LGPS and no experimental or computational reports on LGPO are available to date. Given the growing interest in the mechanical properties of superionic conductors for all-solid-state-battery technologies, justified by the urgency of controlling the manufacturing of these materials and the mechanical phenomena taking place in the electrochemical cell upon cycling (e.g., dendrite propagation through the electrolyte, or formation of interface strains due to volume changes or ionic transport^10,13,14,17^), we believe that further computational and experimental investigations are warranted. Last, whilst elastic properties, like compliance vs. stiffness or ductility vs. brittleness, are governed by the elastic moduli (bulk, shear, Young’s modulus, and Poisson’s ratio), even mechanical properties outside the elastic regime, such as fracture toughness^24,104^, fragility^104^, shear strength^108^, shear viscosity^104^ or hardness^109^, can often be predicted starting from the elastic moduli, underscoring the importance of accurate measurements or predictions.

## Methods

### Elastic tensors from strain fluctuations, and statistical errors

We present here the formalism to calculate elastic tensors from the molecular dynamics simulations according to the strain-fluctuation method^60^, and the derivation of the statistical errors from the dynamics. The strain-stress relation can be recast in terms of the fluctuations of the strain in a constant stress ensemble^60^:

3 $${S}_{\alpha \beta \mu \nu }={\left(\frac{\partial {\epsilon }_{\alpha \beta }}{\partial {\sigma }_{\mu \nu }}\right)}_{T}=\frac{\left\langle V\right\rangle }{{k}_{{{{\rm{B}}}}}T}{\left\langle {{\Delta }}{\epsilon }_{\alpha \beta }{{\Delta }}{\epsilon }_{\mu \nu }\right\rangle }_{N,\sigma ,T}.$$

In Equation (3), *S*_*αβμν*_ is the *αβμν* component of the isothermal compliance tensor^60^ (inverse of the isothermal stiffness tensor {*C*_*αβμν*_}), where *ϵ*_*αβ*_ and *σ*_*μν*_ are the strain and stress tensors, respectively, and the Greek indices *αβμν* cover the cartesian coordinates in three dimensions. *T* and $\left\langle V\right\rangle$ are the temperature and average volume of the system in the constant stress and constant-temperature ensemble (*NσT*), $\left\langle ..\right\rangle$ is an ensemble average, and Δ is a deviation from the mean value, i.e., ${{\Delta }}{\epsilon }_{\alpha \beta }={\epsilon }_{\alpha \beta }-\left\langle {\epsilon }_{\alpha \beta }\right\rangle$. Equation (3) can be derived from statistical thermodynamics through the theory of fluctuations in various ensembles^63,110,111^. The strain tensor in Equation (3) can be calculated from the instantaneous and average cell matrices via the expression^60^:

4 $${{{\boldsymbol{\epsilon }}}}=\frac{1}{2}({\left\langle {{{\bf{h}}}}\right\rangle }^{T,-1}{{{\mathcal{G}}}}{\left\langle {{{\bf{h}}}}\right\rangle }^{-1}-1),$$

where ${{{\mathcal{G}}}}={{{{\bf{h}}}}}^{T}{{{\bf{h}}}}$ is the metric tensor (cf. Supplementary Methods) and **h** the instantaneous cell matrix in the triangular superior form^112^:

5 $${{{\bf{h}}}}=\left(\begin{array}{ccc}\parallel {{{\bf{a}}}}\parallel &\parallel {{{\bf{b}}}}\parallel \cos \gamma &\parallel {{{\bf{c}}}}\parallel \cos \beta \\ 0&\parallel {{{\bf{b}}}}\parallel \sin \gamma &({{{\bf{b}}}}\cdot {{{\bf{c}}}}-{b}_{x}\cdot {c}_{x})/{b}_{y}\\ 0&0&{(\parallel {{{\bf{c}}}}{\parallel }^{2}-{c}_{x}^{2}-{c}_{y}^{2})}^{\frac{1}{2}}\end{array}\right)$$

where ∥**a**∥, ∥**b**∥, ∥**c**∥ and *α*, *β*, *γ* are the cell edges and angles, respectively. Equations (3)−(5) can be used to calculate the isothermal stiffness coefficients from molecular dynamics or Monte Carlo simulations from the strain fluctuations at fixed stress^64,112–115^.

In the Voigt notation^92,116^, thanks to symmetry, the stress and strain tensors can be represented as one-dimensional arrays with six components. The strain is:

6 $${\epsilon }_{1}={\epsilon }_{xx},{\epsilon }_{2}={\epsilon }_{yy},{\epsilon }_{3}={\epsilon }_{zz},{\epsilon }_{4}=2{\epsilon }_{yz},{\epsilon }_{5}=2{\epsilon }_{zx},{\epsilon }_{6}=2{\epsilon }_{xy},$$

and the stress–strain relation is simplified, with the stiffness tensor becoming a (6 × 6) matrix^117^:

7 $${\sigma }_{i}={c}_{ij}{\epsilon }_{j},$$

so that Equation (3) becomes:

8 $${c}_{ij}={\left(\frac{\partial {\sigma }_{i}}{\partial {\epsilon }_{j}}\right)}_{T}=\frac{{k}_{{{{\rm{B}}}}}T}{\left\langle V\right\rangle }{\left\langle {{\Delta }}{{{\boldsymbol{\epsilon }}}}{{\Delta }}{{{\boldsymbol{\epsilon }}}}\right\rangle }_{ij}^{-1},$$

where $\left\langle {{\Delta }}{{{\boldsymbol{\epsilon }}}}{{\Delta }}{{{\boldsymbol{\epsilon }}}}\right\rangle$ is the dynamical covariance of ***ϵ***, and the ensemble average is intended to be in the (*NσT*) ensemble. Equation (8) can also be written as:

9 $${c}_{ij}=\frac{{k}_{{{{\rm{B}}}}}T}{\left\langle V\right\rangle }{\left[\left\langle {{{\boldsymbol{\epsilon }}}}{{{\boldsymbol{\epsilon }}}}\right\rangle -\left\langle {{{\boldsymbol{\epsilon }}}}\right\rangle \left\langle {{{\boldsymbol{\epsilon }}}}\right\rangle \right]}_{ij}^{-1}.$$

In the Voigt notation, adopted throughout the remainder of this paper, the compliance reads:

10 $${s}_{ij}=\frac{\left\langle V\right\rangle }{{k}_{{{{\rm{B}}}}}T}\left[\left\langle {\epsilon }_{i}{\epsilon }_{j}\right\rangle -\left\langle {\epsilon }_{i}\right\rangle \left\langle {\epsilon }_{j}\right\rangle \right].$$

Next, we calculate the errors on the statistical quantities $\left\langle V\right\rangle$, $\left\langle {{{\boldsymbol{\epsilon }}}}\right\rangle$, and $\left\langle {{{\boldsymbol{\epsilon \epsilon }}}}\right\rangle$ by performing a block analysis, as reported in Section Results “Elastic tensors and moduli from the strain fluctuations". Then, we propagate these errors (Var(*V*), Var(***ϵ***) and Var(***ϵϵ***)) to the compliance of Equation (10):

11 $$\begin{array}{l}{{{\rm{Var}}}}({s}_{ij})={\left(\frac{\left\langle V\right\rangle }{{k}_{{{{\rm{B}}}}}T}\right)}^{2}\left[{{{\rm{Var}}}}({\epsilon }_{i}{\epsilon }_{j})+\left\langle {\epsilon }_{i}\right\rangle {{{\rm{Var}}}}({\epsilon }_{j})\right.\\\qquad\qquad \quad\,\,+\,\left.\left\langle {\epsilon }_{j}\right\rangle {{{\rm{Var}}}}({\epsilon }_{i})\right]+{\left(\frac{{s}_{ij}}{\left\langle V\right\rangle }\right)}^{2}{{{\rm{Var}}}}(V)\end{array}$$

and we obtain the error on the stiffness, Var(**c**), by the following:

12 $$\begin{array}{ll}{{{\rm{Var}}}}({{{\bf{c}}}})&={{{\rm{Var}}}}({{{{\bf{s}}}}}^{-1})={d}^{2}\left({{{{\bf{s}}}}}^{-1}\right)={\left({{{{\bf{s}}}}}^{-1}d{{{\bf{s}}}}{{{{\bf{s}}}}}^{-1}\right)}^{2}={\left\{{{{{\bf{s}}}}}_{ij}^{-1}d{s}_{jk}{{{{\boldsymbol{s}}}}}_{km}^{-1}\right\}}^{2}\\& =\left\{{{{{\bf{s}}}}}_{ij}^{-1}d{s}_{jk}{{{{\bf{s}}}}}_{km}^{-1}{{{{\bf{s}}}}}_{il}^{-1}d{s}_{ln}{{{{\bf{s}}}}}_{nm}^{-1}\right\}\\ &=\left\{{{{{\bf{s}}}}}_{ij}^{-1}{{{{\bf{s}}}}}_{ij}^{-1}d{s}_{jk}d{s}_{jk}{{{{\bf{s}}}}}_{km}^{-1}{{{{\bf{s}}}}}_{km}^{-1}\right\}+{\left\{o\left(d{s}_{jk}d{s}_{ln}\right)\right\}}_{j\ne l,k\ne n}\\ &=\left\{{({{{{\bf{s}}}}}_{ij}^{-1})}^{2}{{{\rm{Var}}}}({s}_{jk}){({{{{\bf{s}}}}}_{km}^{-1})}^{2}\right\},\end{array}$$

where, in the last passage, we exclude the term ${\left\{o\left(d{s}_{jk}d{s}_{ln}\right)\right\}}_{j\ne l,k\ne n}$ as we consider *s*_*ij*_ to be statistically decorrelated. We note that the compliance and stiffness tensors computed with this procedure are independent of the number of blocks chosen for the error analysis.

### Elastic tensors from static approaches

We provide here a concise overview of the static methods to evaluate elastic constants and moduli. The expression for the cell matrix under the strain ***ϵ*** is:

13 $$\left(\begin{array}{rcl}{{{{\bf{a}}}}}_{{{{\bf{1}}}}}^{{\prime} }&{{{{\bf{a}}}}}_{{{{\bf{2}}}}}^{{\prime} }&{{{{\bf{a}}}}}_{{{{\bf{3}}}}}^{{\prime} }\end{array}\right)=(I+{{{\boldsymbol{\epsilon }}}})\left(\begin{array}{rcl}{{{{\bf{a}}}}}_{{{{\bf{1}}}}}&{{{{\bf{a}}}}}_{{{{\bf{2}}}}}&{{{{\bf{a}}}}}_{{{{\bf{3}}}}}\end{array}\right)=\left(\begin{array}{rcl}1+{\epsilon }_{1}&{\epsilon }_{6}/2&{\epsilon }_{5}/2\\ {\epsilon }_{6}/2&1+{\epsilon }_{2}&{\epsilon }_{4}/2\\ {\epsilon }_{5}/2&{\epsilon }_{4}/2&1+{\epsilon }_{3}\end{array}\right)\left(\begin{array}{rcl}{{{{\bf{a}}}}}_{{{{\bf{1}}}}}&{{{{\bf{a}}}}}_{{{{\bf{2}}}}}&{{{{\bf{a}}}}}_{{{{\bf{3}}}}}\end{array}\right)$$

where $$\left(\begin{array}{rcl}{{{{\bf{a}}}}}_{{{{\bf{1}}}}}^{{\prime} }&{{{{\bf{a}}}}}_{{{{\bf{2}}}}}^{{\prime} }&{{{{\bf{a}}}}}_{{{{\bf{3}}}}}^{{\prime} }\end{array}\right)$$ and $$\left(\begin{array}{rcl}{{{{\bf{a}}}}}_{{{{\bf{1}}}}}&{{{{\bf{a}}}}}_{{{{\bf{2}}}}}&{{{{\bf{a}}}}}_{{{{\bf{3}}}}}\end{array}\right)$$ are the strained and unstrained cells, respectively. Under a hydrostatic strain

14 $$\begin{array}{lll}{\epsilon }_{i}&\!\!\!=x,i&\!\!\!=1,2,3;\\ {\epsilon }_{i}&\!\!\!=0,i&\!\!\!=4,5,6,\end{array}$$

only the volume *V* of the cell is changed, and the bulk modulus (or isothermal incompressibility, i.e., the stiffness of the material under the effect of isotropic compression) can be calculated fitting an EOS, such as Murnaghan’s^118,119^, Birch’s^119,120^, Keane’s^97,121^, or Stacey’s^122^, where *P* and *V* are the variables, and volume, bulk modulus, and bulk modulus derivatives at the minimum (*V*_0_, *B*_0_, ${B}_{0}^{{\prime} }$, ${B}_{0}^{{\prime\prime} }$.) are the fitting parameters. We use here a simple Murnaghan’s EOS, equivalent to Keane’s EOS^97,98,121^ with ${B}_{0}^{{\prime\prime} }=0$^85,98^, where the energy-volume relation is:

15 $$E=-\frac{{B}_{0}{V}_{0}}{{B}_{0}^{{\prime} }-1}+\frac{V{B}_{0}}{{B}_{0}^{{\prime} }}\left[{\left(\frac{{V}_{0}}{V}\right)}^{{B}_{0}^{{\prime} }}\frac{1}{({B}_{0}^{{\prime} }-1)}+1\right]$$

and the pressure

16 $$P=\frac{{B}_{0}}{{B}_{0}^{{\prime} }}\left[{\left(\frac{V}{{V}_{0}}\right)}^{-{B}_{0}^{{\prime} }}-1\right].$$

While the bulk modulus can be obtained directly from the EOS (Equations (15) and (16)), the remaining moduli (shear, Young’s and Poisson’s ratio) can only be derived from the full elastic tensor (Equation (7)), with 21 independent components^92^. Symmetry reduces this number to, e.g., 13 for monoclinic, 9 for orthorhombic, and 6 for tetragonal space groups^92^. The full elastic tensor can be calculated by an “energy-strain" approach^53^, i.e., by expanding the energy over the strain up to the second order^50,123^, or by a “stress–strain" approach, based on the stress resulting from an applied strain^51,54^. In the stress–strain approach, the elastic tensor is calculated from the change in stress Δ***σ*** associated with the strain ***ϵ*** applied to a reference configuration ***ϵ***_0_^51^:

17 $${{\Delta }}{{{\boldsymbol{\sigma }}}}={{{\boldsymbol{\sigma }}}}({{{{\boldsymbol{\epsilon }}}}}_{{{{\bf{0}}}}}+{{{\boldsymbol{\epsilon }}}})-{{{\boldsymbol{\sigma }}}}({{{{\boldsymbol{\epsilon }}}}}_{0})={{{\bf{c}}}}({{{{\boldsymbol{\epsilon }}}}}_{{{{\bf{0}}}}}){{{\boldsymbol{\epsilon }}}}.$$

We recall that in Equation (17) ***σ*** and ***ϵ*** are six-dimensional vectors and **c**(***ϵ***_**0**_) is a (6 × 6) matrix (Voigt notation^116^). The whole tensor **c**(***ϵ***_**0**_) can be obtained from Equation (17) by imposing the 6 simple uniaxial strains

18 $$\begin{array}{l}{{{{\boldsymbol{\epsilon }}}}}_{m}=\{{\epsilon }_{i}\},\\ {\epsilon }_{i}={\delta }_{im}x,m=1,\ldots 6.\end{array}$$

From Equations (17) and (18), each column *m* of **c**(***ϵ***_**0**_) is extracted from the differences between the stress calculated at ***ϵ***_**0**_ + ***ϵ***_*m*_ and ***ϵ***_**0**_^51^:

19 $${{\Delta }}{{{{\boldsymbol{\sigma }}}}}_{m}={{{\boldsymbol{\sigma }}}}({{{{\boldsymbol{\epsilon }}}}}_{{{{\bf{0}}}}}+{{{{\boldsymbol{\epsilon }}}}}_{m})-{{{\boldsymbol{\sigma }}}}({{{{\boldsymbol{\epsilon }}}}}_{0})=\left({c}_{1m},{c}_{2m},{c}_{3m},{c}_{4m},{c}_{5m},{c}_{6m}\right)x.$$

This is a simple and general way to extract the elastic tensor from the stress–strain relations. The 36 stiffness coefficients can be obtained independently, although only 21 are necessary (as **c**(***ϵ***_**0**_) is a symmetric matrix). The method holds for the most general case of a triclinic phase^51^, i.e., the independent matrix elements can be in principle all different. Of course, **c**(***ϵ***_**0**_) should display the symmetries of the space group to which the materials belong, which can also become a useful checkpoint for the convergence of the calculations.

### From the elastic tensors to the elastic moduli: Voigt-Reuss-Hill approximation

The effective elastic moduli of an arbitrarily shaped isotropic polycrystalline aggregate can be obtained from the elastic stiffness tensor {*c*_*ij*_} of the single crystal, by assuming homogeneous strain (Voigt approximation^116^):

20 $$\begin{array}{ll}9{B}_{{{{\rm{V}}}}}&={c}_{11}+{c}_{22}+{c}_{33}+2({c}_{12}+{c}_{23}+{c}_{31})\\ 15{G}_{{{{\rm{V}}}}}&={c}_{11}+{c}_{22}+{c}_{33}-({c}_{12}+{c}_{23}+{c}_{31})\\ &\quad\,+\,3({c}_{44}+{c}_{55}+{c}_{66}),\end{array}$$

or from the elastic compliance tensor {*s*_*ij*_}, by assuming homogeneous stress (Reuss approximation^124^):

21 $$\begin{array}{ll}1/{B}_{{{{\rm{R}}}}}&={s}_{11}+{s}_{22}+{s}_{33}+2({s}_{12}+{s}_{23}+{s}_{31})\\ 15/{G}_{{{{\rm{R}}}}}&=4({s}_{11}+{s}_{22}+{s}_{33})-4({s}_{12}+{s}_{23}+{s}_{31})\\ &\quad\,\,+\,3({s}_{44}+{s}_{55}+{s}_{66}).\end{array}$$

Equations (20) and (21) constitute the upper and lower bounds to the expected values of the moduli, respectively^125,126^, and their arithmetic or geometric means are considered a reasonable estimate, which goes under the name of Voigt-Reuss-Hill (VRH) approximation^126^:

22 $$\begin{array}{lll}B&\!\!\!=\frac{1}{2}({B}_{{{{\rm{V}}}}}+{B}_{{{{\rm{R}}}}})\,{{{\rm{or}}}}\,\,B&\!\!\!=\sqrt{{B}_{{{{\rm{V}}}}}{B}_{{{{\rm{R}}}}}},\\ G&\!\!\!=\frac{1}{2}({G}_{{{{\rm{V}}}}}+{G}_{{{{\rm{R}}}}})\,{{{\rm{or}}}}\,\,G&\!\!\!=\sqrt{{G}_{{{{\rm{V}}}}}{G}_{{{{\rm{R}}}}}}.\end{array}$$

In Equations (20) and (21) only the 9 independent components of the elastic tensors of orthorhombic single crystals appear^92^. A more sophisticated approach would be to relax the isotropy and homogeneity assumptions, using the variational principle that holds for arbitrary crystal shapes^127^. The resulting bounds, which go under the name of Hashin-Shtrikman bounds, have been derived for cubic^128^, orthorhombic^129^, tetragonal^130^, and monoclinic^131^ symmetries, and prove to give more accurate results than the VRH approximation^128–131^, though still lying within the Voigt and the Reuss bounds^128,131^. Thus, for simplicity, and considering the scope of this work, we will restrict ourselves here to the VRH approximation. Young’s modulus *E* and Poisson’s ratio *ν* are calculated from *B* and *G* through the relations^126^:

23 $$\frac{1}{E}=\frac{1}{3G}+\frac{1}{9B},\quad \nu =\frac{1}{2}\left[1-\frac{3G}{3B+G}\right].$$

For the strain-fluctuation method, where a statistical error is provided for the elastic tensors (see above), the errors on the elastic moduli *B*, *G*, *E,* and Poisson’s ratio are obtained by propagating the errors on the compliance (Equation (11)) and the stiffness (Equation (12)) to the expressions of the moduli in the VRH approximation (Equations (20) and (23))^116,124,126^.

### Supplementary information

Supplementary Information: Solids that are also liquids: Elastic tensors of superionic materials

## Data Availability

All relevant computational results and data are provided in the Materials Cloud repository^132^.

## Code availability

Generation of the trajectories was performed with the open-source code QUANTUM ESPRESSO^85^. The open-source code following the described protocol to extract the elastic moduli and their statistical errors from the trajectories can be found at https://github.com/materzanini.
