# Supplementary material for: Solids that are also liquids: elastic tensors of superionic materials
Source: NPJ Comput Mater. 2023 Jan 19;9(1):10. doi: 10.1038/s41524-022-00948-8 (PMC11041723; doi:10.1038/s41524-022-00948-8)
Supplement: Supplementary file 1 — Supplementary Information: Solids that are also liquids: Elastic tensors of superionic materials [file 41524_2022_948_MOESM1_ESM.pdf]

Supplementary Information

Solids that are also liquids: Elastic tensors of superionic materials

Giuliana Materzanini,[1,2,*] Tommaso Chiarotti,[1,2] and Nicola Marzari[1,2]

[1] *Theory and Simulations of Materials (THEOS),*

École Polytechnique Fédérale de Lausanne, CH-1015 Lausanne, Switzerland

[2] *National Centre for Computational Design and Discovery of Novel Materials (MARVEL),*

École Polytechnique Fédérale de Lausanne, CH-1015 Lausanne, Switzerland

* Corresponding author: giuliana.materzanini@uclouvain.be; Present address: Institute of Condensed Matter and Nanosciences, Université Catholique de Louvain, B-1348 Louvain-la-Neuve, Belgium.

I. SUPPLEMENTARY METHODS

In the following, we give a concise overview of the basic methods in isobaric-isothermal Car-Parrinello simulations. In classical molecular dynamics, for a system of N atoms with masses $\{M_I\}$, positions $\{\mathbf{R}_I\}$ and an interaction potential ϕ, the isobaric-isothermal ensemble can be generated from the Parrinello-Rahman (PR) lagrangian [1, 2] with a Nose-Hoover thermostat [3]:

$$\begin{aligned}\mathcal{L}^{\mathrm{PR}}_{\mathrm{classic}} = \frac{1}{2}\sum_I^N M_I(\dot{\mathbf{S}}_I^\dagger \mathcal{G}\dot{\mathbf{S}}_I) + \frac{1}{2}Q\dot{\xi}^2 + \frac{1}{2}W\mathrm{Tr}(\dot{\mathbf{h}}^\dagger\dot{\mathbf{h}}) - \phi(\{\mathbf{h}\ \mathbf{S}_I\},\mathbf{h}) + \\ -(3N+1)k_BTln\xi - P\det[\mathbf{h}].\end{aligned} \tag{1}$$

In addition to the microcanonical lagrangian, $\mathcal{L}^{\mathrm{PR}}_{\mathrm{classic}}$ contains the kinetic energies of a thermostat with mass Q and a barostat with mass W, and their interactions with the atomic system. The time dependent variables are the atomic scaled positions $\mathbf{S}_I = \mathbf{h}^{-1}\mathbf{R}_I$, where $\mathcal{G} = \mathbf{h}^\dagger\mathbf{h}$ is the metric tensor, the generalized variable for thermostat motion ξ, and the cell matrix $\mathbf{h}$, being $\det[\mathbf{h}] = V$ the volume of the cell. While the Nose-Hoover thermostat keeps the temperature constant at T by rescaling the atomic velocities [3], the barostat keeps the pressure constant at P while scaling the atomic coordinates $\{\mathbf{R}_i\}$ by the time-dependent cell vectors $\mathbf{h}$. In Car-Parrinello (CP) molecular dynamics, one can either thermostat only the ions or include an additional thermostat for the electrons [4], and we note the barostat scales not only the ionic but also the electronic coordinates $\mathbf{s} = \mathbf{h}^{-1}\mathbf{r}$ [5]. The CP lagrangian, without thermostatting the electrons [4], reads [5, 6]:

$$\begin{aligned}\mathcal{L}^{\mathrm{PR}}_{\mathrm{CP}} = \frac{1}{2}\sum_I^N M_I(\dot{\mathbf{S}}_I^\dagger \mathcal{G}\dot{\mathbf{S}}_I) + \frac{1}{2}Q\dot{\xi}^2 + \frac{1}{2}W\mathrm{Tr}(\dot{\mathbf{h}}^\dagger\dot{\mathbf{h}}) + \mu\sum_i\int d\mathbf{s}|\dot{\psi}_i(\mathbf{s})|^2 + \\ -E^{CP}[\{\psi_i\},\{\mathbf{h}\ \mathbf{S}_I\}] - (3N+1)k_BTln\xi - P\det[\mathbf{h}].\end{aligned} \tag{2}$$

In Eq. (2) the mass associated to the electronic wavefunctions μ has to be chosen to ensure adiabaticity, also dependently on the choice of the time step, as in the genuine CP method [7–9], and the masses of the thermostat (Q) and barostat (W) are chosen in such a way that their associated frequencies ω_t and ω_b are of the order of magnitude of the frequency of a sound wave travelling the nearest neighbour distance and the cell length, respectively [6, 10, 11]:

$$\omega_t = \frac{\sqrt{k_B T}}{Q}; \tag{3}$$

$$\omega_b = \frac{\sqrt{k_B T}}{W}. \tag{4}$$

In Eq. (4), ω_b can be directly recast to the total mass of the cell M through the relation [12]:

$$W = \frac{0.75M}{2\pi}. \tag{5}$$

In the following, we give some details of the isobaric-isothermal Car-Parrinello simulations presented in the main text. We employ a plane-wave cutoff of $E_{cut} = 50$ Ry and ultrasoft pseudopotentials (with 400 Ry cutoff for the electron density) chosen from version 1.1 of the Standard Solid State Pseudopotential (SSSP) Efficiency library [13, 14], namely GBRV [15] for Li, O and S, and PSLib [16] for Ge and P. Brillouin zone integrations are performed using the Γ point, and the exchange-correlation functional is PBE [17], in accordance with the pseudopotential chosen. In Ref. [9] we showed the **k**-point convergence for the relaxed geometries of these cells (Table I of Ref. [9]). However, since the scope of the present simulations is to compute elastic tensors, we show here also the convergence of the stress tensor and pressure over the **k**-point grid. We choose two uncorrelated snapshots from the dynamics reported above, having very low and very high value of pressure, respectively. For each of them, we perform two SCF calculations, one at the Γ point and one at an unshifted (2, 2, 2) **k**-point grid [18]. We perform these tests on the two benchmark systems t-LGPS and o-LGPO, and report the results in Supplementary Tables 7 and 8, respectively. The maximum deviation is 1×10^{-2} GPa and 2×10^{-2} GPa for the pressure, and 4×10^{-2} GPa and 8×10^{-2} GPa for the off-diagonal elements of the stress tensor, for t-LGPS and o-LGPO, respectively. Based on these tests, we conclude that Γ-point sampling is sufficient for the scope of this work. Based on the extended tests done in [9], we choose an electronic mass of 500 a. u., a time step of 4 a. u.(~ 0.1 fs). We choose a thermostat frequency of 17 THz

with a thermostat for each atomic species as done in [9], and a barostat mass proportional to the mass of the system [10, 12], according to Eqs. 4 and 5. However, since the aim of this work is a quantitative study of the cell fluctuations, we additionally perform short NPT simulations for the two benchmark materials t-LGPS and o-LGPO, using barostat masses half and twice the chosen mass. We report the fluctuations of the cell parameters from these simulations in Supplementary Figures 3 and 4 for t-LGPS and o-LGPO, respectively, showing that the fluctuations are very similar in a wide range of barostat masses around the chosen mass [10, 12].

In the following, detailed results from the cell dynamics, in particular for o-LGPS, t-LGPO, and o-LGPO, are reported. In Supplementary Figure 1 we report the orthorhombic cell of 100 atoms used for o-LGPO (Supplementary Figure 1a) and the tetragonal cell of 50 atoms used for t-LGPO (Supplementary Figure 1b), following Ref. [9]. The sulfides o-LGPS and t-LGPS have analogous structures. The adequacy of the supercell sizes is tested both through the study of the stress convergence over the **k**-point grid, described above, and through the calculation of the elastic moduli from the dynamics for t-LGPS in a double-sized (100-atom) supercell, that is discussed below. The values of the cell edges $\|\mathbf{a}\|$, $\|\mathbf{b}\|$, and $\|\mathbf{c}\|$ for the four structures from the 600K-NPT CP trajectory are reported in Fig. 1 of the main text. In Supplementary Figure 2 the cell angles α, β and γ are reported, from the same simulations. We note that the spread of the angles is more pronounced for the sulfide (Supplementary Figures 2a,b) than for the oxide (Supplementary Figures 2c,d), and for the tetragonal phases (Supplementary Figures 2a,c) than for the orthorhombic ones (Supplementary Figures 2b,d). In Supplementary Figure 5 we report the components of the strain $\boldsymbol{\epsilon}(t)$, that we extract from $\|\mathbf{a}\|$, $\|\mathbf{b}\|$, $\|\mathbf{c}\|$, α, β and γ, and in turn from the cell dynamics $\mathbf{h}(t)$ (Eqs. (4)$-$(6) in the main text), for each of the four structures. In Supplementary Figure 6 we report isosurfaces of Li-ion probability density for o-LGPS ($\rho(\mathrm{Li}) = 6 \times 10^{-2}$ Å^{-3}), t-LGPO ($\rho(\mathrm{Li}) = 1 \times 10^{-1}$ Å^{-3}), and o-LGPO ($\rho(\mathrm{Li}) = 4 \times 10^{-2}$ Å^{-3}) from the 600K-NPT CP dynamics. The procedure for extracting the Li-ion probability density from the trajectory is described elsewhere [19] (see also https://github.com/lekah/samos).

In the following, the error determination and the convergence of the moduli for o-LGPS, t-LGPO, and o-LGPO are reported and analyzed. In Supplementary Figure 7 we report the

relative variance of B, G, E, and ν (obtained from error propagation, see Section Results "Elastic tensors and moduli from the strain fluctuations" and Section Methods in the main text) as a function of the number of data in block chosen for the block analysis of $\langle V \rangle$, $\langle \boldsymbol{\epsilon} \rangle$, and $\langle \boldsymbol{\epsilon\epsilon} \rangle$, for o-LGPS, t-LGPO, and o-LGPO (600K-*NPT* CP dynamics). The analogous plot for t-LGPS is shown in Fig. 3 in the main text. By increasing the number of blocks (going right to left in the plot), the variance oscillates less strongly, and reaches a tiny region of stability, after which it starts to decrease monotonically. This plateau determines the proper number of data in block [20] and thus the error. The convergence of the elastic moduli on the simulation time is shown in Supplementary Figure 8 for o-LGPS, t-LGPO, and o-LGPO. Analogous plots, and a description of these calculations at different trajectory lengths, are reported for t-LGPS in Fig. 4 in the main text. For o-LGPS and o-LGPO the errors of B, G, E, and ν decrease, and their absolute values converge, while increasing the simulation time. A similar behaviour is shown by t-LGPS (Fig. 4 in the main text).
For the hypothetical t-LGPO material (Supplementary Figure 8b), the errors are systematically large. Moreover, B, G, E, and ν show always a flat trend, as opposed to the oscillatory trend shown by the other phases, that flattens asymptotically. In addition, the values of the moduli for t-LGPO are significantly lower than the respective moduli for o-LGPO (Supplementary Figure 8c). We investigate the dependence of the elastic constants on the oscillations of the cell by using a (2 x 2 x 1) supercell, already used in [9], that we simulate for 100 ps. In Supplementary Table 6 we report the values of cell parameters and their standard deviations both for the 50-atom and 200-atoms supercells, showing that the amplitude of the oscillations is noticeably diminished by increasing the supercell size. This is also illustrated in Supplementary Figure 9. In Supplementary Table 6, we also report the average values of the moduli from the same simulations through the strain-fluctuation method, showing that decreasing the amplitude of the oscillations has the net effect of increasing the moduli. However, as shown from the high values of the statistical errors on the moduli in Supplementary Table 6, the convergence over the simulation time is still not sufficient, and we conclude that for this material, that shows a facile $a \rightleftharpoons b$ swap, we would need larger supercells, and longer simulation times, to assess its elastic moduli from the strain-fluctuation method.

In the following, finite-size effects on the elastic moduli from the strain-fluctuation method

are tested and discussed. In order to investigate the effects of the size of the cell on the moduli, we simulate t-LGPS in the 100-atom 2 x 1 x 1 cell obtained by doubling the cell reported in Supplementary Figure 1b along a. These simulations are performed for 70 ps at 600 K. The results for the moduli and statistical errors as a function of the trajectory length (obtained following the same procedure described in Section Results "Elastic tensors and moduli from the strain fluctuations" and Section Methods in the main text) are reported in Supplementary Figure 10 (light colours) together with the analogous results for the 1 x 1 x 1 cell (dark colours, see also Fig. 4 in the main text). Although the moduli and their statistical errors converge more slowly for the larger cell, the limited simulation time ($\sim$ 70 ps) employed for this test simulation already shows an excellent agreement between the 1 x 1 x 1 and the 2 x 1 x 1 cells for E and G, and for ν within the statistical errors. The agreement is less satisfactory for B, for which we record a 15% discrepancy between the two cells, that we ascribe to the fact that the size of the cell has been increased anisotropically. This effect is related to the change of the average volume from the dynamics between the two cells, reported in Supplementary Table 1a, and to the behaviour of the volume obtained after variable-cell relaxation at different **k**-point samplings, that we report in Supplementary Table 1b: going from Γ to (2, 2, 2), (3, 3, 3), and (4, 4, 4) **k**-point grids, we observe a nice convergence pattern, whereas the calculations with anisotropic (2,1,1), (1,2,1), and (1,1,2) grids give values outside this pattern. It is not surprising that, among the moduli, the effect of this anisotropic increasing of the cell is seen only on B, which is directly related to the volume [21]. On the other hand, these simulations show that choosing a 2 x 2 x 2 cell (400 atoms) wouldn't be computationally affordable, as the convergence of the moduli over time worsens significantly by increasing the size of the simulation cell (Supplementary Figure 10). Overall, these test results (Supplementary Figure 10) show that our calculations are not significantly affected by finite-size effects.

In the following, we present the temperature dependence of the elastic moduli for o-LGPO. In Supplementary Figure 11, the lattice parameters of the o-LGPO phase are reported as a function of the temperature (T =600, 800, and 1000 K), as reported in Fig. 5 of the main text for t-LGPS. In Supplementary Figure 12, the values of B, G, E, and ν of o-LGPO as a function of temperature are reported, together with a fit to the Wachtman's law [22–24],

as reported in Fig. 6 of the main text for t-LGPS.

In the following, we report more details on the static calculations of the elastic moduli for t-LGPS. In Supplementary Figure 13 we report energy-volume calculations for t-LGPS on the global minimum energy structure [25], performed by keeping the atoms fixed at their equilibrium positions, together with the corresponding fit to the Murnaghan EOS (Eq. (15) in the main text). For a comparison, we also report the results from the corresponding calculations performed with internal coordinate relaxation, as in Fig. 7 of the main text. The bulk modulus from the unrelaxed-atoms calculations (B^*(EOS) = 48.0 GPa) is more than twice as large as the one from the relaxed-atoms calculations (B(EOS) = 21.7 GPa), as reported in Table 4 in the main text. Since the latter is much more in line with experimental data [26] and with the results from the strain-fluctuation method presented in this work, we conclude that for these systems relaxing the internal coordinates is crucial to obtain reliable moduli. In Supplementary Tables 2, 3, and 4 we report the full elastic tensors and Voigt-Reuss bounds for the moduli from the stress-strain calculations on nine fully relaxed snapshots chosen from the dynamics. In Supplementary Table 5 we report the same results on the global minimum energy structure from Ref. [25].

II. SUPPLEMENTARY FIGURES

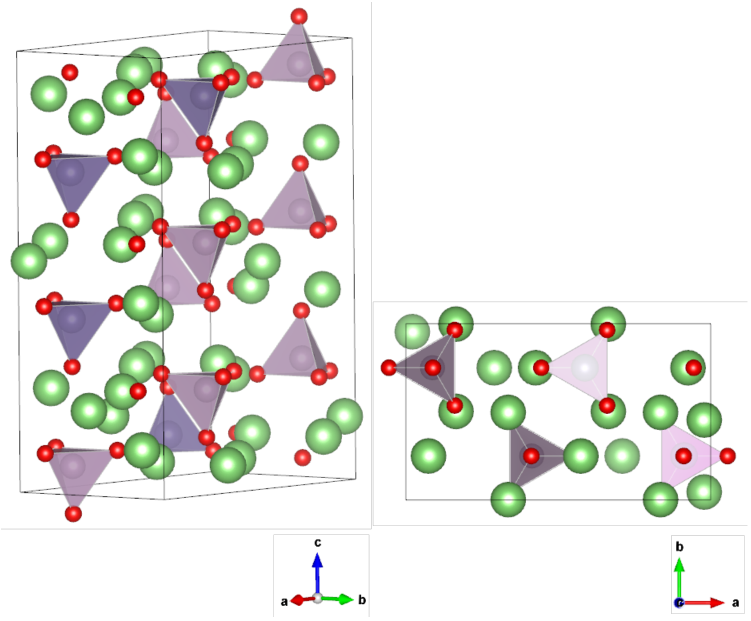

(a)

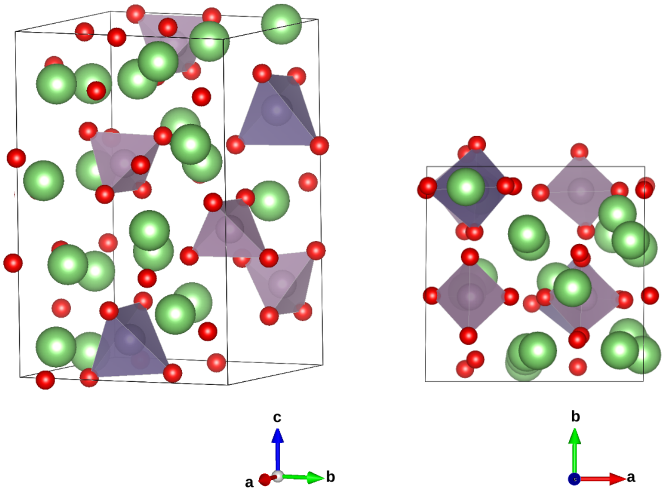

(b)

Supplementary Figure 1: The 100-atom and 50-atom supercells (side and top view) used in the simulations for (a) o-LGPO and (b) t-LGPO from Refs. [27] and [28], respectively (see also Refs. [9, 29]). Li atoms are displayed in green, O atoms are in red, and Ge and P atoms are at the center of the dark and light purple tetrahedra, respectively. The analogous LGPS supercells have sulphur atoms replacing oxygen atoms. From Ref. [9].

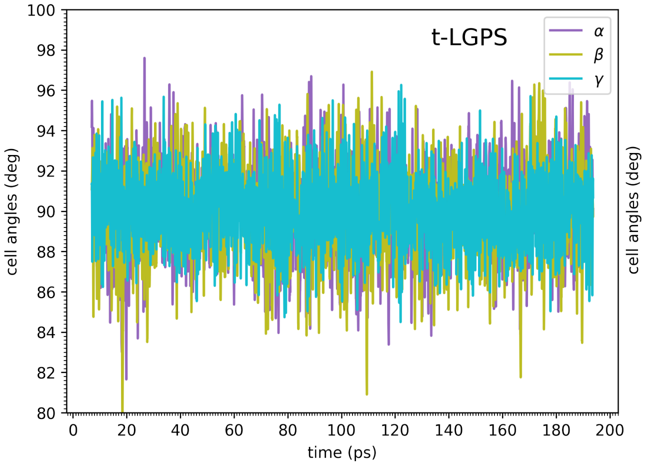

(a) t-LGPS

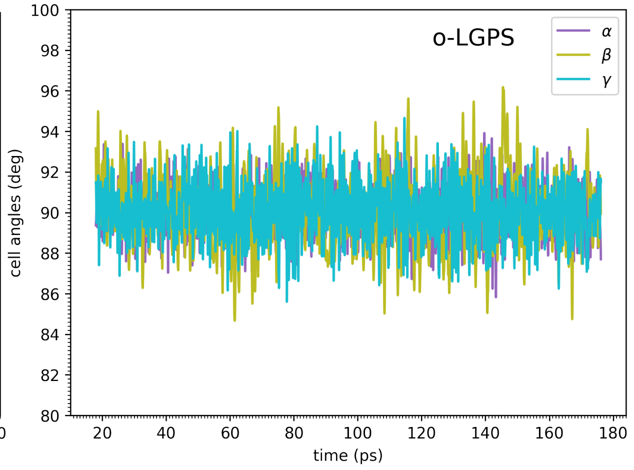

(b) o-LGPS

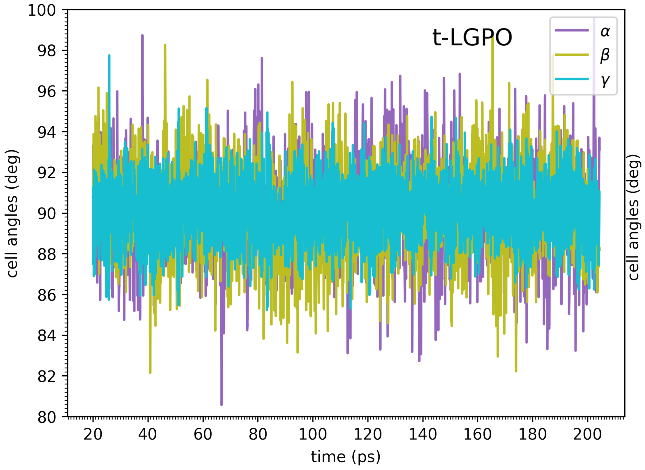

(c) t-LGPO

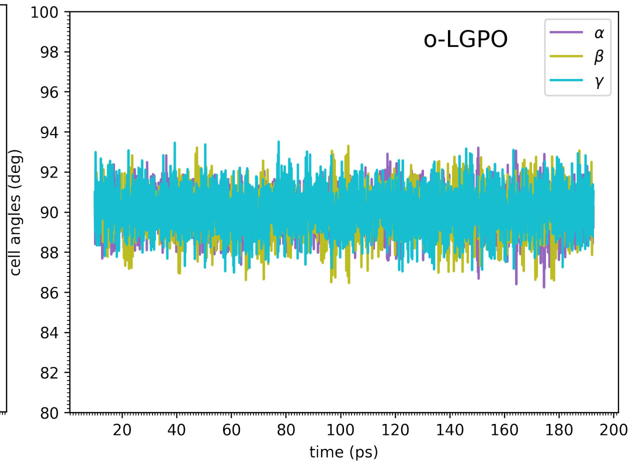

(d) o-LGPO

Supplementary Figure 2: Cell angles (α, β and γ) in the 600K-NPT CP molecular dynamics for the four structures studied.

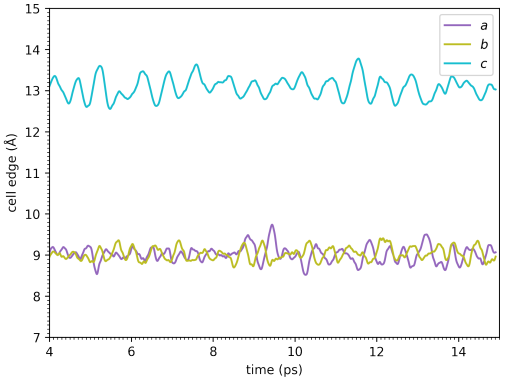

(a)

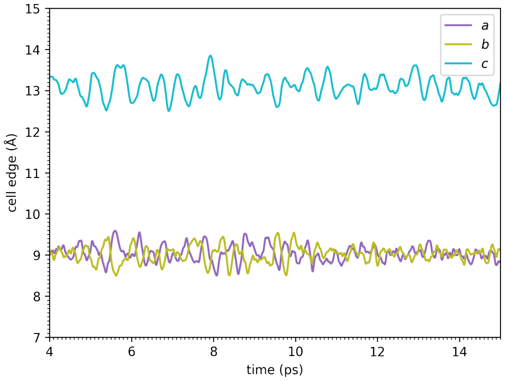

(b)

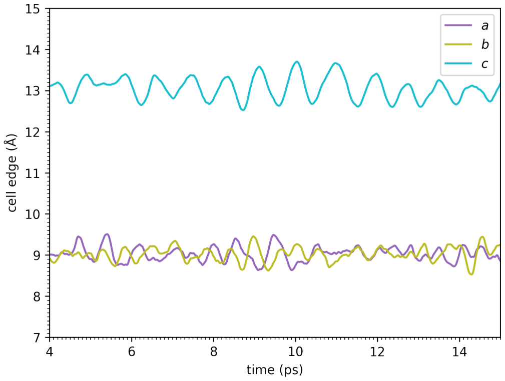

(c)

Supplementary Figure 3: Dependence of the cell parameter fluctuations on the mass of the barostats for t-LGPS: a) with W from Eq. (5); b) with W half as in a); c) with W twice as in a).

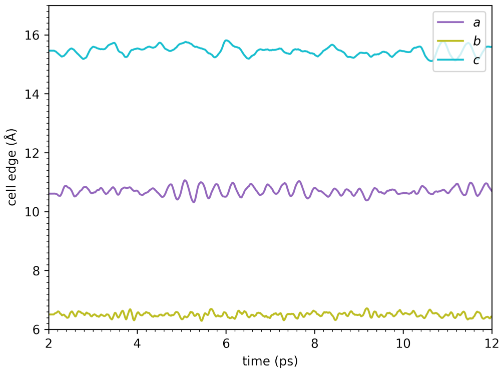

(a)

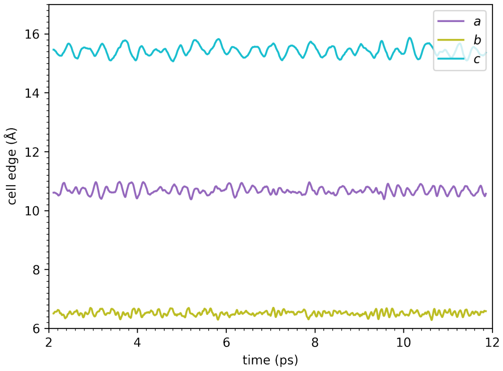

(b)

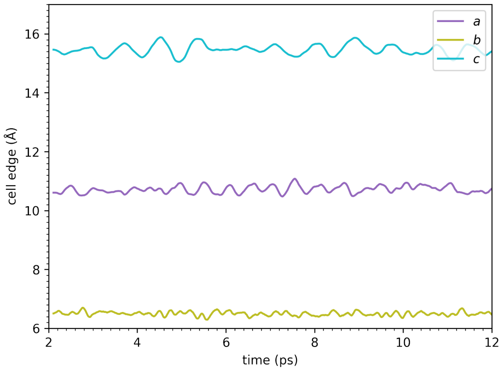

(c)

Supplementary Figure 4: Dependence of the cell parameter fluctuations on the mass of the barostats for o-LGPO: a) with W from Eq. (5); b) with W half as in a); c) with W twice as in a).

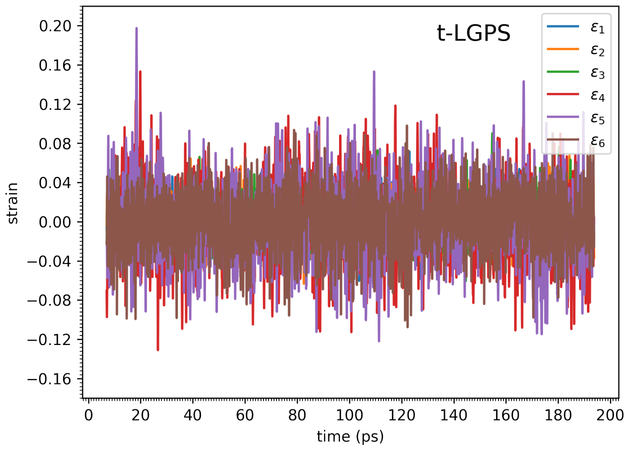

(a) t-LGPS

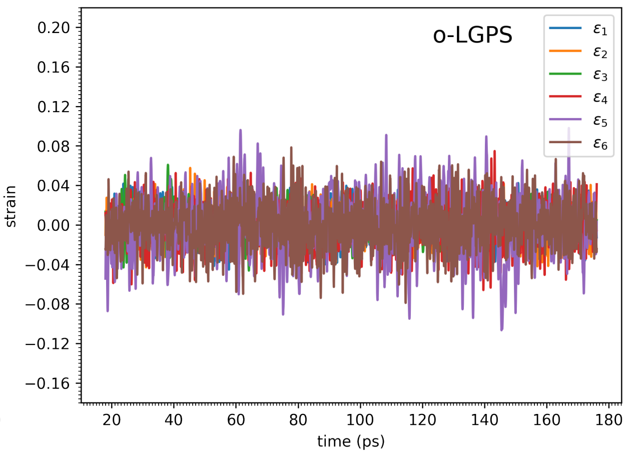

(b) o-LGPS

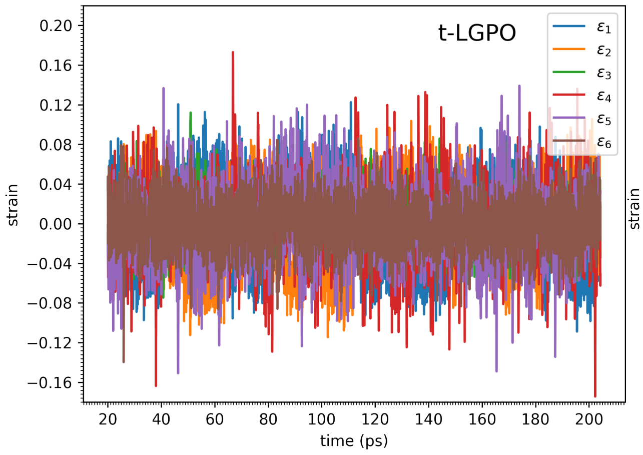

(c) t-LGPO

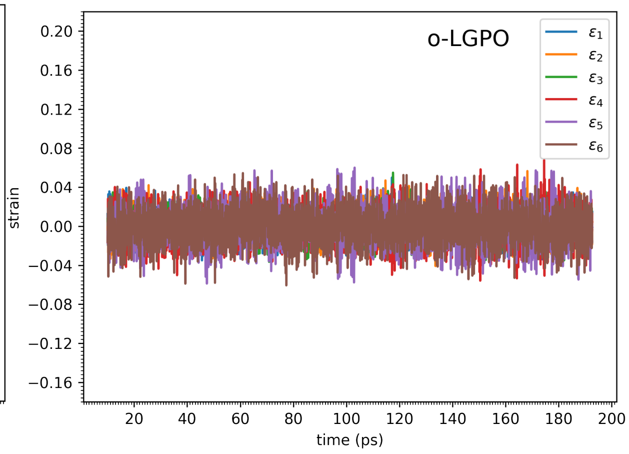

(d) o-LGPO

Supplementary Figure 5: Components of the strain in Voigt notation, calculated from the 600K-NPT CP molecular dynamics (Eqs. (4)−(6) in the main text), for the four structures studied. In the plots, the same range for $\epsilon_i(t)$ is used.

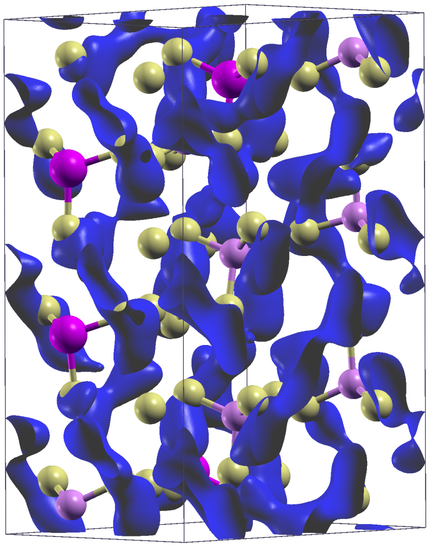

(a) o-LGPS

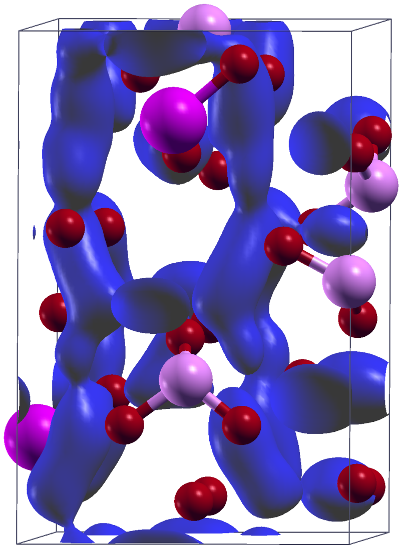

(b) t-LGPO

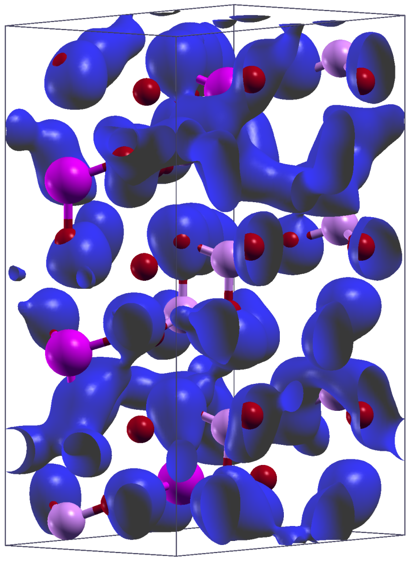

(c) o-LGPO

Supplementary Figure 6: Li-ion probability density in o-LGPS, t-LGPO, and o-LGPO from 600K-*NPT* CP molecular dynamics trajectories. The Li-ion density isovalues (blue isosurfaces) are 0.06, 0.1, and 0.04 $Å^{-3}$ for o-LGPS, t-LGPO, and o-LGPO, respectively. The equilibrium positions of oxygen (sulfur), germanium, and phosphorus are shown as red (yellow), pink, and light rose spheres, respectively, and Ge−S, Ge−O, P−S, and P−O bonds are displayed.

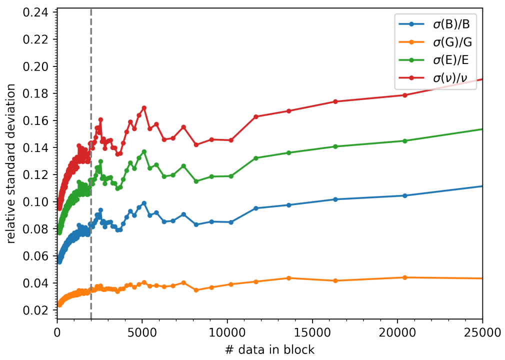

(a) o-LGPS

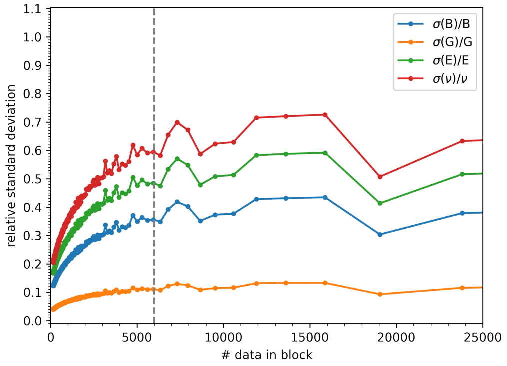

(b) t-LGPO

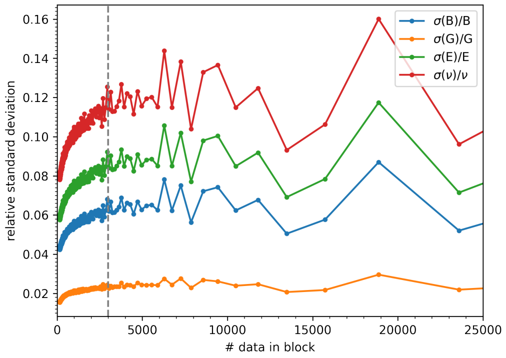

(c) o-LGPO

Supplementary Figure 7: Relative standard deviation of B, G, E, and ν (cf. Fig. 3 in the main text) as a function of the number of data in block used to calculate the variance of $\langle V \rangle$, $\langle \boldsymbol{\epsilon} \rangle$, and $\langle \boldsymbol{\epsilon\epsilon} \rangle$ (see Section Results "Elastic tensors and moduli from the strain fluctuations" and Method Section in the main text), from the 600K-NPT CP molecular dynamics of o-LGPS, t-LGPO, and o-LGPO. In each plot, the first point on the right corresponds to 4 blocks, and the maximum number of blocks considered is 600. Based on these plots, we have chosen 41, 16, and 31 blocks for the error block analysis for o-LGPS, t-LGPO, and o-LGPO, respectively. Our choice is reported in the figure by the vertical dashed lines (each block is $\sim$ 4 ps long in (a), $\sim$ 10 ps long in (b), and $\sim$ 5 ps long in (c)).

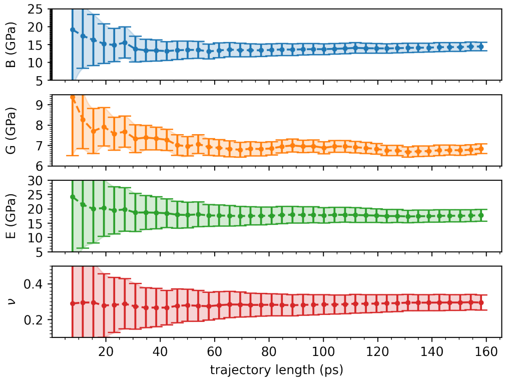

(a) o-LGPS

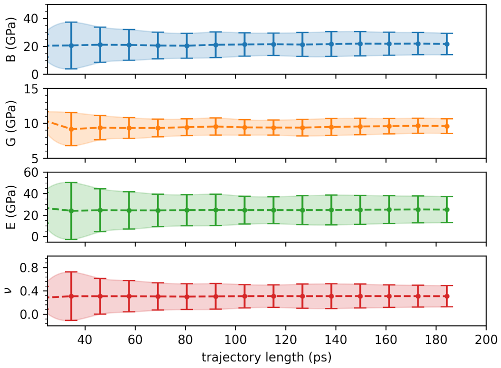

(b) t-LGPO

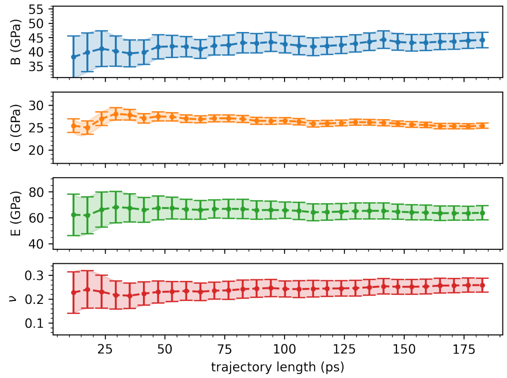

(c) o-LGPO

Supplementary Figure 8: Dependence of the elastic moduli and Poisson's ratio on the length of the 600K-NPT CP molecular dynamics trajectory for o-LGPS, t-LGPO, and o-LGPO. Each point corresponds to a trajectory which is n-block long, with $n = 2, ..N$ blocks, N being the number of blocks that we have chosen for the whole trajectory (see Supplementary Figure 7), each block containing the # data as determined from Supplementary Figure 7. The error bars are the standard errors of the moduli for each trajectory, obtained from the variance over the blocks, since these blocks are already uncorrelated and there is no need to repeat the block analysis in Supplementary Figure 7 for each of these calculations. The analogous plots for t-LGPS are reported in Fig. 4 of the main text.

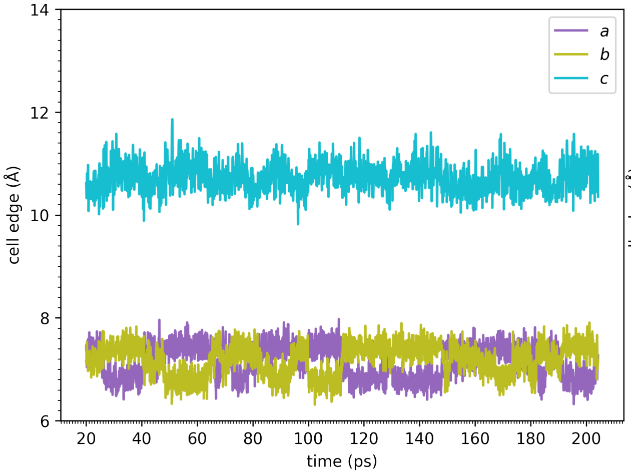

(a)

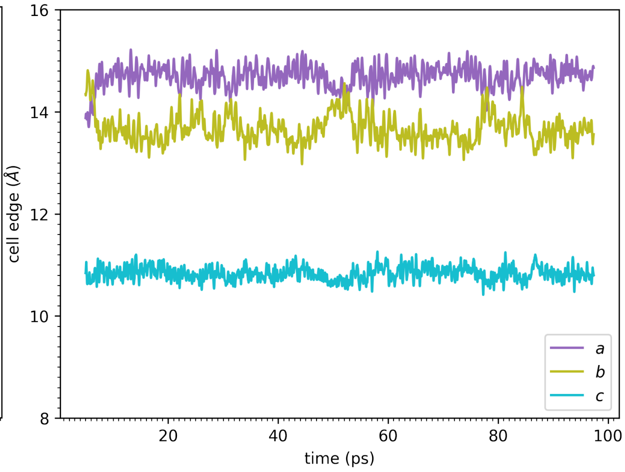

(b)

Supplementary Figure 9: Dependence of the cell oscillations on the size of the supercell for t-LGPO: a) lattice parameters for the 50-atom supercell; b) same as a) for the 200-atom supercell. The cell oscillations are considerably smoothed (the relative statistical uncertainty is halved, see also Supplementary Table 6) when increasing the size of the cell by 4 times.

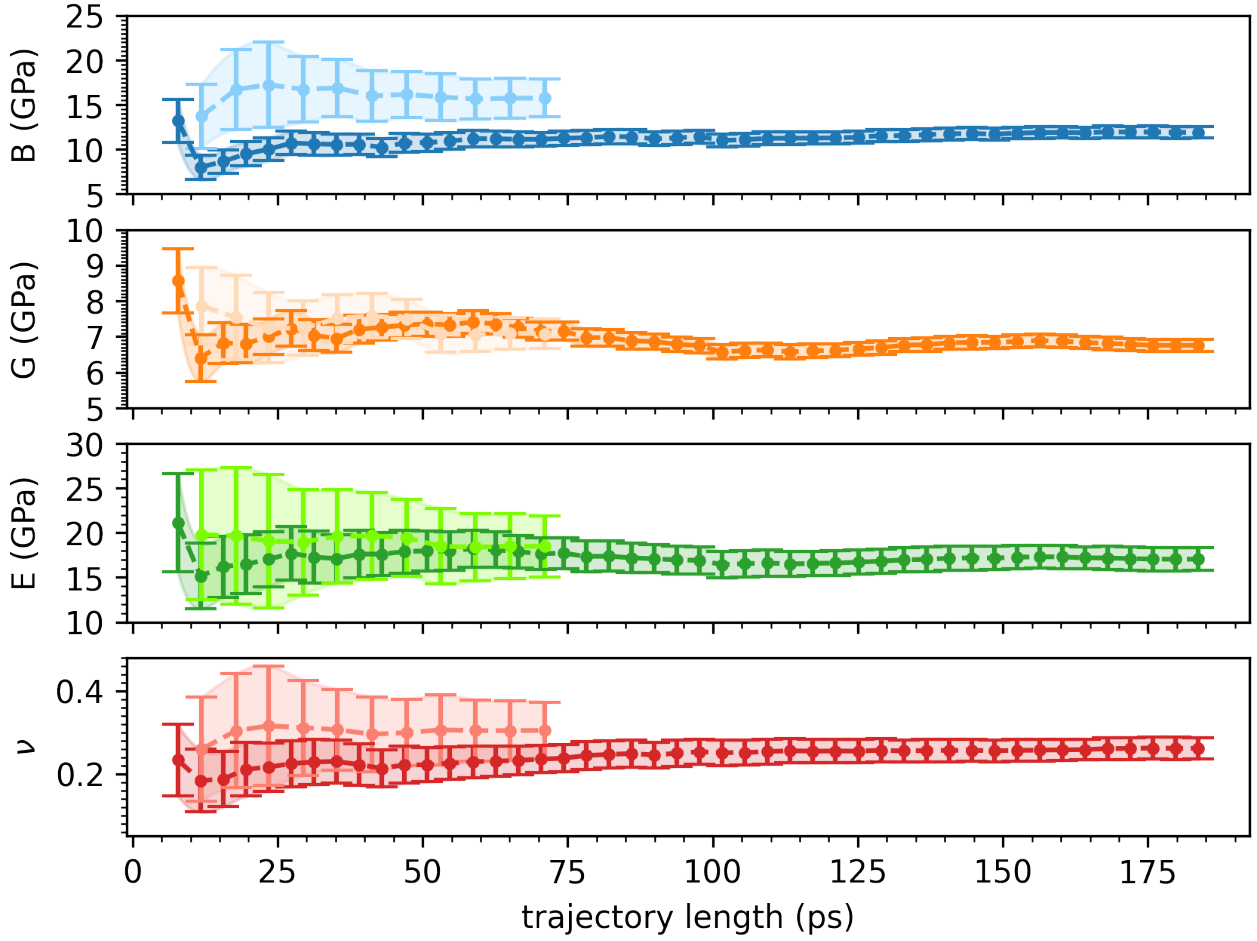

Supplementary Figure 10: Dependence of the elastic moduli and Poisson's ratio on the length of the 600K-*NPT* CP molecular dynamics trajectory for t-LGPS simulated in a 2 x 1 x 1 supercell (light colours). For a comparison, we also report (dark colours) the analogous results for the 1 x 1 x 1 cell of Supplementary Figure 1, from Fig. 4 of the main text. For the error bars description, see Supplementary Figure 8. We conclude that the elastic moduli in the 1 x 1 x 1 cell do not suffer significanty from finite-size effects. See text for an explanatory discussion.

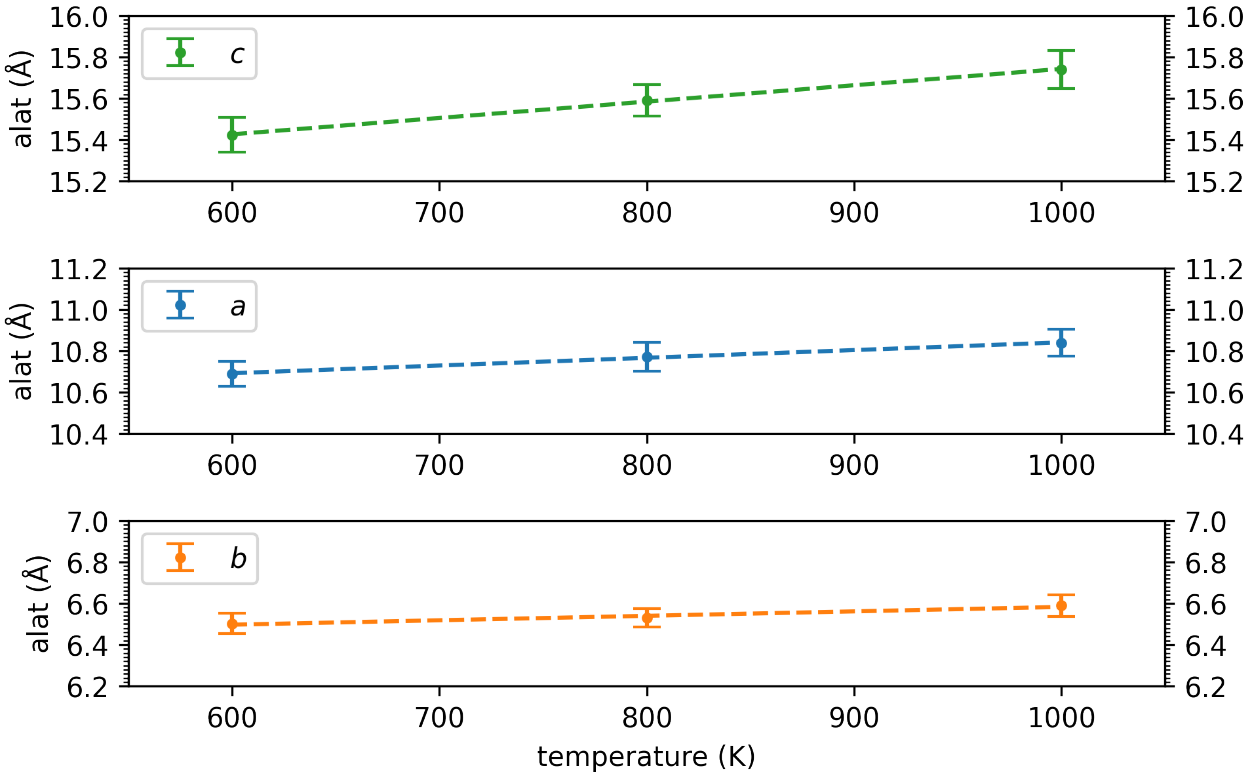

Supplementary Figure 11: Temperature dependence of the o-LGPO lattice parameters. The error bars are the standard deviations over the blocks, from a block analysis performed for each lattice parameter, as shown in Fig. 7c for the elastic moduli.

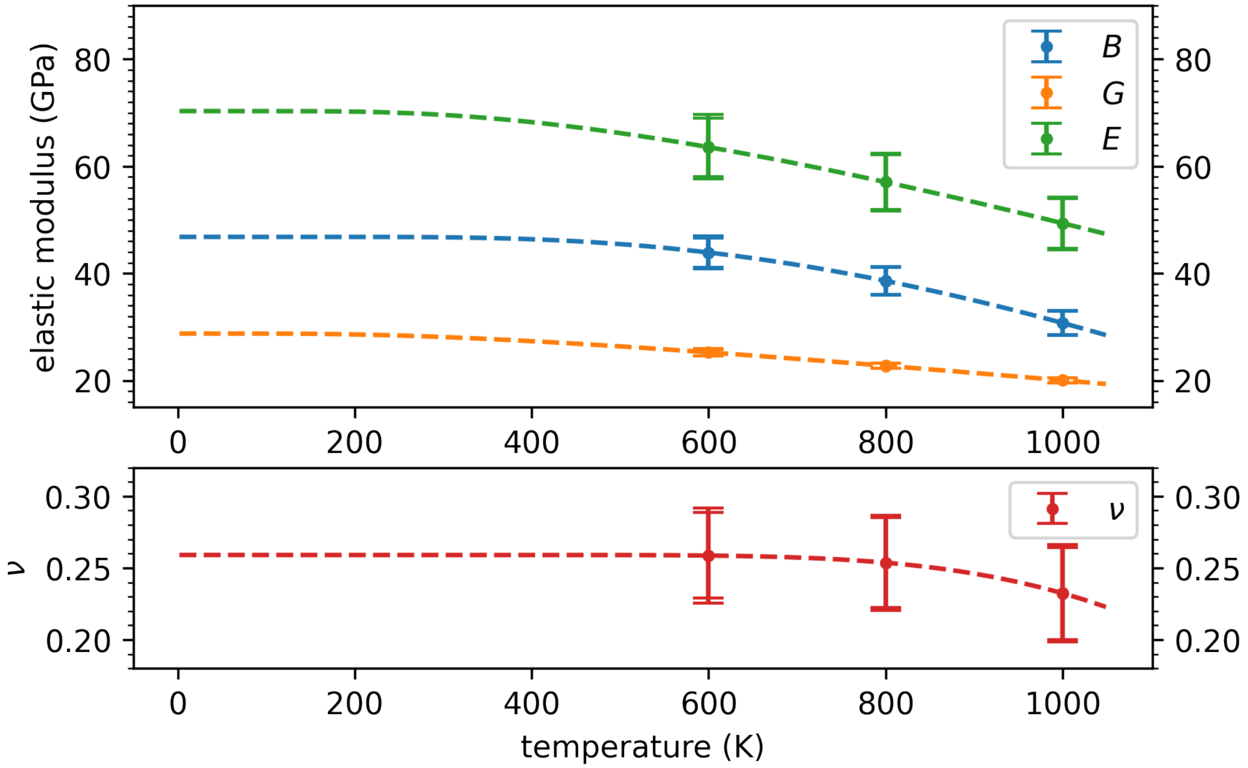

Supplementary Figure 12: Temperature dependence of B, G, E, and ν for o-LGPO from NPT CP molecular dynamics at 600, 800, and 1000 K. The error bars are the standard errors of the moduli over the trajectories, after a block analysis as from Supplementary Figure 7. The computed data are fitted to the Wachtman's equation (main text and Refs. [22–24]). An analogous plot for t-LGPS is reported in Fig. 5 in the main text.

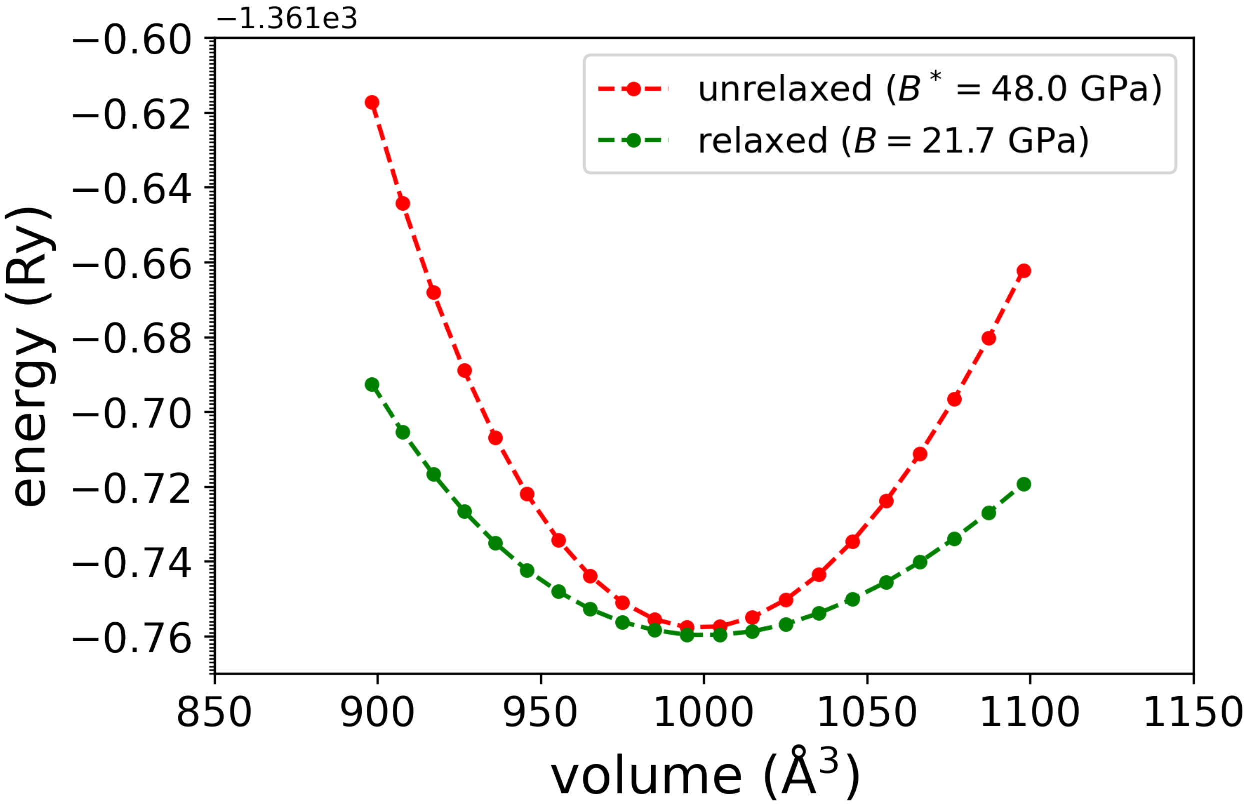

Supplementary Figure 13: Comparison between the energy-volume curves for the atoms in the global energy minimum configuration [25] fixed at their equilibrium positions (red points) and relaxed (green points). The dotted lines are fits to the Murnaghan EOS (Eq. (15) in the main text). The bulk modulus from the unrelaxed-atoms calculations (B^*(EOS) = 48.0 GPa) is more than twice as large as the one from the relaxed-atoms calculations (B(EOS) = 21.7 GPa), as reported in Table 4 in the main text.

III. SUPPLEMENTARY TABLES

a) t-LGPS: 600K-NPT simulations	
cell size	average volume (Å^3)
1 x 1 x 1 (50 atoms)	1060.88 ± 1.61
2 x 1 x 1 (100 atoms)	2105.57 ± 1.77

b) t-LGPS: fully relaxed calculations	
**k**-point sampling	volume (Å^3)
Γ	996.10
$(2, 1, 1)$	989.31
$(1, 2, 1)$	1000.07
$(1, 1, 2)$	995.23
$(2, 2, 2)$	998.44
$(3, 3, 3)$	998.17
$(4, 4, 4)$	998.17

Supplementary Table 1: a) Convergence of the average volume on the supercell size from the molecular dynamics 600K-NPT simulations for t-LGPS, showing a contraction of the cell when the cell size is doubled along one dimension. b) Convergence of the volume on the **k**-point sampling (unshifted grids [18]) from the fully relaxed calculations for t-LGPS, showing that doubling the **k**-point grid only in one dimension produces an overall error higher than the Γ sampling.

configuration	elastic tensor	B_V(GPa)<br>B_R(GPa)<br>B(GPa)	G_V(GPa)<br>G_R(GPa)<br>G(GPa)	E_V(GPa)<br>E_R(GPa)<br>E(GPa)	ν_V(GPa)<br>ν_R(GPa)<br>ν(GPa)
Snapshot#1	$\begin{pmatrix} 40.4 & 15.8 & 11.1 & 1.9 & 0.9 & -0.6 \\ 16.0 & 42.5 & 10.7 & 0.1 & 1.7 & 0.6 \\ 11.4 & 10.6 & 35.9 & 0.5 & 2.9 & -0.3 \\ 1.8 & 0.1 & 0.6 & 8.0 & -0.2 & 1.4 \\ 1.0 & 1.7 & 2.8 & -0.0 & 9.9 & 1.4 \\ -0.8 & 0.6 & -0.1 & 1.8 & 1.4 & 16.2 \end{pmatrix}$	21.62<br>20.85<br>21.23	12.21<br>10.42<br>11.31	30.82<br>26.80<br>28.81	0.26<br>0.29<br>0.27
Snapshot#2	$\begin{pmatrix} 40.3 & 15.7 & 10.2 & -1.0 & 1.8 & -1.4 \\ 16.5 & 42.3 & 11.5 & -0.1 & 1.4 & -1.6 \\ 10.5 & 10.9 & 37.8 & -3.0 & 2.9 & 0.8 \\ -1.0 & -0.3 & -3.1 & 11.4 & -0.0 & 1.1 \\ 1.8 & 1.3 & 2.9 & -0.0 & 8.8 & -0.7 \\ -1.3 & -1.4 & 0.7 & 1.0 & -0.6 & 16.2 \end{pmatrix}$	21.75<br>19.06<br>20.40	12.78<br>12.72<br>12.75	32.07<br>31.21<br>31.64	0.25<br>0.23<br>0.24
Snapshot#3	$\begin{pmatrix} 42.1 & 17.6 & 11.2 & -0.1 & -0.5 & 2.3 \\ 16.5 & 46.4 & 11.4 & 0.5 & -0.6 & 0.7 \\ 11.1 & 14.9 & 35.1 & -1.1 & -1.8 & 0.5 \\ 0.4 & 4.5 & -1.1 & 12.1 & 0.4 & -0.4 \\ -0.4 & 5.3 & -1.6 & 0.4 & 10.1 & -0.2 \\ 2.9 & 1.8 & 0.8 & -0.5 & -0.2 & 16.4 \end{pmatrix}$	22.92<br>20.60<br>21.76	13.21<br>13.15<br>13.18	33.24<br>32.52<br>32.88	0.26<br>0.24<br>0.25

Supplementary Table 2: Elastic stiffness tensors and moduli (Voigt-Reuss bounds and VRH average) for t-LGPS from the stress-strain method on fully relaxed snapshots from the dynamics, where the internal coordinates are relaxed at each distorsion. Snapshots #1−#3.

configuration	elastic tensor	B_V(GPa) B_R(GPa) B(GPa)	G_V(GPa) G_R(GPa) G(GPa)	E_V(GPa) E_R(GPa) E(GPa)	ν_V(GPa) ν_R(GPa) ν(GPa)
Snapshot#4	$\begin{pmatrix} 40.4 & 15.8 & 10.3 & -1.3 & -0.7 & 1.2 \\ 16.2 & 41.0 & 11.0 & 0.3 & -1.1 & 1.5 \\ 10.6 & 10.6 & 37.4 & -3.3 & -3.5 & -0.6 \\ -1.2 & 0.2 & -3.0 & 10.0 & 0.3 & -0.8 \\ -0.7 & -1.1 & -3.4 & 0.3 & 10.5 & -0.7 \\ 1.1 & 1.6 & -0.6 & -0.8 & -0.8 & 17.0 \end{pmatrix}$	21.48<br>19.62<br>20.55	12.96<br>11.98<br>12.47	32.37<br>29.87<br>31.12	0.25<br>0.25<br>0.25
Snapshot#5	$\begin{pmatrix} 40.2 & 15.8 & 10.7 & 1.7 & 0.8 & -0.4 \\ 16.0 & 41.7 & 10.3 & -0.1 & 1.6 & 0.7 \\ 11.3 & 10.5 & 35.9 & 0.7 & 3.0 & -0.3 \\ 1.6 & 0.0 & 0.6 & 7.9 & -0.1 & 1.4 \\ 0.9 & 1.6 & 2.9 & -0.0 & 9.9 & 1.3 \\ -0.7 & 0.7 & -0.1 & 1.7 & 1.3 & 15.7 \end{pmatrix}$	21.38<br>20.59<br>20.99	12.08<br>10.37<br>11.23	30.50<br>26.64<br>28.57	0.26<br>0.28<br>0.27
Snapshot#6	$\begin{pmatrix} 44.1 & 17.1 & 11.0 & -2.8 & -0.2 & 0.4 \\ 17.5 & 45.0 & 12.1 & 0.3 & -0.6 & 0.8 \\ 11.0 & 12.1 & 37.9 & -4.0 & -1.1 & 0.1 \\ -2.7 & 0.3 & -4.0 & 12.1 & 0.3 & 0.0 \\ -0.1 & -0.7 & -1.1 & 0.3 & 7.2 & -1.4 \\ 0.4 & 0.8 & 0.1 & -0.0 & -1.6 & 15.7 \end{pmatrix}$	23.11<br>20.25<br>21.68	12.78<br>13.19<br>12.98	32.37<br>32.51<br>32.44	0.27<br>0.23<br>0.25

Supplementary Table 3: Elastic stiffness tensors and moduli (Voigt-Reuss bounds and VRH average) for t-LGPS from the stress-strain method on fully relaxed snapshots from the dynamics, where the internal coordinates are relaxed at each distorsion. Snapshots #4−#6.

configuration	elastic tensor	B_V(GPa)<br>B_R(GPa)<br>B(GPa)	G_V(GPa)<br>G_R(GPa)<br>G(GPa)	E_V(GPa)<br>E_R(GPa)<br>E(GPa)	ν_V(GPa)<br>ν_R(GPa)<br>ν(GPa)
Snapshot#7	$\begin{pmatrix} 41.1 & 18.6 & 11.4 & -0.2 & -0.4 & -1.3 \\ 18.2 & 36.6 & 11.7 & 0.4 & 0.7 & -0.7 \\ 11.1 & 10.9 & 35.7 & -0.6 & -0.0 & -0.8 \\ -0.3 & -0.1 & -0.8 & 12.4 & -0.6 & 0.2 \\ -0.1 & 1.4 & 0.1 & -0.6 & 9.7 & -1.0 \\ -1.2 & -0.1 & -1.2 & 0.2 & -1.1 & 18.5 \end{pmatrix}$	21.71<br>21.94<br>21.82	12.94<br>13.13<br>13.04	32.39<br>32.84<br>32.62	0.25<br>0.25<br>0.25
Snapshot#8	$\begin{pmatrix} 42.3 & 17.5 & 11.1 & -0.6 & 0.5 & 0.5 \\ 17.5 & 38.8 & 11.3 & 0.2 & -1.0 & 1.1 \\ 11.1 & 11.0 & 39.1 & -1.3 & -0.9 & 1.2 \\ -0.7 & -0.1 & -1.3 & 9.1 & -0.0 & -0.7 \\ 0.6 & -0.9 & -0.9 & -0.1 & 11.7 & -0.4 \\ 0.6 & 1.7 & 1.5 & -0.9 & -0.4 & 16.3 \end{pmatrix}$	22.20<br>22.48<br>22.34	12.79<br>11.56<br>12.17	32.19<br>29.60<br>30.89	0.26<br>0.28<br>0.27
Snapshot#9	$\begin{pmatrix} 44.4 & 16.2 & 11.9 & 1.8 & -0.5 & 0.2 \\ 16.6 & 37.7 & 10.1 & -0.2 & -0.3 & -0.4 \\ 12.0 & 9.9 & 40.9 & 0.3 & -1.1 & -0.2 \\ 1.4 & 0.0 & 0.5 & 7.7 & 0.1 & -0.0 \\ -0.5 & -0.3 & -1.0 & 0.7 & 11.1 & 0.7 \\ 0.4 & -0.4 & -0.2 & -0.1 & 0.7 & 16.3 \end{pmatrix}$	22.18<br>24.71<br>23.45	12.66<br>10.76<br>11.71	31.91<br>28.20<br>30.05	0.26<br>0.31<br>0.29

Supplementary Table 4: Elastic stiffness tensors and moduli (Voigt-Reuss bounds and VRH average) for t-LGPS from the stress-strain method on fully relaxed snapshots from the dynamics, where the internal coordinates are relaxed at each distorsion. Snapshots #7−#9.

configuration	elastic tensor	B_V(GPa)<br>B_R(GPa)<br>B(GPa)	G_V(GPa)<br>G_R(GPa)<br>G(GPa)	E_V(GPa)<br>E_R(GPa)<br>E(GPa)	ν_V(GPa)<br>ν_R(GPa)<br>ν(GPa)
t−LGPS<br>0K (fully optimized)<br>[25]	$\begin{pmatrix} 45.4 & 17.4 & 11.5 & -3.0 & -0.1 & 0.4 \\ 17.3 & 44.0 & 12.1 & 0.3 & -0.7 & 0.8 \\ 10.8 & 12.0 & 36.0 & -4.3 & -1.1 & 0.1 \\ -3.2 & 0.4 & -4.3 & 12.5 & 0.3 & 0.2 \\ -0.1 & -0.7 & -1.0 & 0.4 & 10.4 & -1.5 \\ 0.4 & 0.8 & 0.2 & 0.2 & -1.8 & 15.3 \end{pmatrix}$	22.93<br>20.49<br>21.7	13.31<br>13.21<br>13.3	33.46<br>32.63<br>33.0	0.26<br>0.23<br>0.25

Supplementary Table 5: Elastic stiffness tensors and moduli (Voigt-Reuss bounds and VRH average) for t-LGPS from the stress-strain method on the global minimum energy structure (here fully relaxed) from Ref. [25]. The internal coordinates are relaxed at each distorsion.

	50-atom cell (200 ps)	200-atom cell (100 ps)
$a(\AA)$	7.15 ± 0.22	14.69 ± 0.15
$b(\AA)$	7.19 ± 0.16	13.66 ± 0.18
$c(\AA)$	10.74 ± 0.15	10.83 ± 0.12
B (GPa)	21.65 ± 7.57	31.07 ± 18.76
G (GPa)	9.58 ± 1.04	13.27 ± 2.83
E (GPa)	25.05 ± 11.9	34.84 ± 28.93
ν	0.31 ± 0.18	0.31 ± 0.31

Supplementary Table 6: Dependence of average lattice parameters a, b, c and elastic moduli obtained from the strain-fluctuation method on the size of the supercell for t-LGPO. The relative statistical uncertainty of the lattice parameters is halved (see also Supplementary Figure 9), and the values of the moduli are considerably increased. However, the statistical uncertainties on the moduli increase, due to the shorter length of the trajectory.

	$\mathbf{k}$ points: Γ	$\mathbf{k}$ points: (2 x 2 x 2)
stress tensor (GPa) (low-P snapshot)	$\begin{pmatrix} -0.212 & 1.062 & -0.068 \\ 1.062 & -1.706 & -0.240 \\ -0.068 & -0.240 & 0.433 \end{pmatrix}$	$\begin{pmatrix} -0.083 & 1.094 & -0.090 \\ 1.094 & -1.870 & -0.261 \\ -0.090 & -0.261 & 0.513 \end{pmatrix}$
P (GPa) (low-P snapshot)	-0.495	-0.480
stress tensor (GPa) (high-P snapshot)	$\begin{pmatrix} 3.278 & 0.152 & -0.295 \\ 0.152 & 1.337 & -0.629 \\ -0.295 & -0.629 & 1.216 \end{pmatrix}$	$\begin{pmatrix} 3.185 & 0.145 & -0.286 \\ 0.145 & 1.306 & -0.666 \\ -0.286 & -0.666 & 1.380 \end{pmatrix}$
P (GPa) (high-P snapshot)	-1.944	-1.957

Supplementary Table 7: Convergence of the stress tensor and pressure on the **k**-point grid (Γ and (2 x 2 x 2)) for t-LGPS, showing maximum deviations of 10^{-2} GPa for the pressure, and 4×10^{-2} GPa for the off-diagonal elements of the stress tensor.

	$\mathbf{k}$ points: Γ	$\mathbf{k}$ points: (2 x 2 x 2)
stress tensor (GPa)<br>(low-P snapshot)	$\begin{pmatrix} -0.448 & -0.459 & -0.324 \\ -0.459 & -0.616 & -0.475 \\ -0.324 & -0.475 & -1.363 \end{pmatrix}$	$\begin{pmatrix} -0.006 & -0.467 & -0.303 \\ -0.467 & -1.454 & -0.477 \\ -0.303 & -0.477 & -1.045 \end{pmatrix}$
P (GPa)<br>(low-P snapshot)	-0.809	-0.835
stress tensor (GPa)<br>(high-P snapshot)	$\begin{pmatrix} 2.357 & 0.568 & -1.660 \\ 0.568 & 1.974 & 0.969 \\ -1.660 & 0.969 & 1.338 \end{pmatrix}$	$\begin{pmatrix} 2.866 & 0.568 & -1.647 \\ 0.568 & 0.882 & 0.992 \\ -1.647 & 0.992 & 1.690 \end{pmatrix}$
P (GPa)<br>(high-P snapshot)	1.890	-1.813

Supplementary Table 8: Convergence of the stress tensor and pressure on the $\mathbf{k}$-point grid (Γ and (2 x 2 x 2)) for o-LGPO, showing maximum deviations of 2×10^{-2} GPa for the pressure, and 8×10^{-2} GPa for the off-diagonal elements of the stress tensor.

IV. SUPPLEMENTARY REFERENCES

[1] Parrinello, M. & Rahman, A. Crystal structure and pair potentials: A molecular-dynamics study. *Phys. Rev. Lett.* **45**, 1196 (1980).

[2] Parrinello, M. & Rahman, A. Polymorphic transitions in single crystals: A new molecular dynamics method. *J. Appl. Phys* **52**, 7182–7190 (1981).

[3] Nosé, S. A unified formulation of the constant temperature molecular dynamics methods. *J. Chem. Phys.* **81**, 511–519 (1984).

[4] Blöchl, P. E. & Parrinello, M. Adiabaticity in first-principles molecular dynamics. *Phys. Rev. B* **45**, 9413 (1992).

[5] Bernasconi, M. *et al.* First-principle-constant pressure molecular dynamics. *J Phys Chem Solids* **56**, 501–505 (1995).

[6] Martyna, G. J., Tobias, D. J. & Klein, M. L. Constant pressure molecular dynamics algorithms. *J. Chem. Phys.* **101**, 4177–4189 (1994).

[7] Pastore, G., Smargiassi, E. & Buda, F. Theory of ab initio molecular-dynamics calculations. *Phys. Rev. A* **44**, 6334–6347 (1991).

[8] Galli, G. & Pasquarello, A. First-principles molecular dynamics. In *Computer simulation in chemical physics*, 261–313 (Springer, 1993).

[9] Materzanini, G., Kahle, L., Marcolongo, A. & Marzari, N. High Li-ion conductivity in tetragonal LGPO: A comparative first-principles study against known LISICON and LGPS phases. *Phys. Rev. Mater.* **5**, 035408 (2021).

[10] Martyna, G. J., Klein, M. L. & Tuckerman, M. Nosé–Hoover chains: The canonical ensemble via continuous dynamics. *J. Chem. Phys.* **97**, 2635–2643 (1992).

[11] Nosé, S. & Klein, M. Constant pressure molecular dynamics for molecular systems. *Mol. Phys.* **50**, 1055–1076 (1983).

[12] Giannozzi, P. *et al.* Quantum espresso: a modular and open-source software project for quantum simulations of materials. *J. Phys. Condens. Matter* **21**, 395502 (2009).

[13] Prandini, G., Marrazzo, A., Castelli, I. E., Mounet, N. & Marzari, N. Precision and efficiency in solid-state pseudopotential calculations. *npj Comput. Mater.* **4**, 72 (2018).

[14] Lejaeghere, K. *et al.* Reproducibility in density functional theory calculations of solids. *Science* **351**, aad3000 (2016).

[15] Garrity, K. F., Bennett, J. W., Rabe, K. M. & Vanderbilt, D. Pseudopotentials for high-throughput DFT calculations. *Comput. Mater. Sci.* **81**, 446–452 (2014).

[16] Dal Corso, A. Pseudopotentials periodic table: From H to Pu. *Comput. Mater. Sci.* **95**, 337–350 (2014).

[17] Perdew, J. P., Burke, K. & Ernzerhof, M. Generalized gradient approximation made simple. *Phys. Rev. Lett.* **77**, 3865 (1996).

[18] Monkhorst, H. J. & Pack, J. D. Special points for brillouin-zone integrations. *Phys. Rev. B* **13**, 5188 (1976).

[19] Kahle, L., Marcolongo, A. & Marzari, N. High-throughput computational screening for solid-state Li-ion conductors. *Energy Environ. Sci.* **13**, 928–948 (2020).

[20] Frenkel, D. & Smit, B. *Understanding molecular simulation: from algorithms to applications*, vol. 1 (Elsevier, 2001).

[21] Nye, J. F. *et al. Physical properties of crystals: their representation by tensors and matrices* (Oxford university press, 1985).

[22] Wachtman Jr, J., Tefft, W., Lam Jr, D. & Apstein, C. Exponential temperature dependence of Young's modulus for several oxides. *Phys. Rev.* **122**, 1754 (1961).

[23] Anderson, O. L. Derivation of Wachtman's equation for the temperature dependence of elastic moduli of oxide compounds. *Phys. Rev.* **144**, 553 (1966).

[24] Rajagopalan, S. On the validity of modified Wachtman's equation for nonoxide solids. *Phys. Status Solidi B* **40**, 513–516 (1970).

[25] Ong, S. P. *et al.* Phase stability, electrochemical stability and ionic conductivity of the $Li_{10\pm1}MP_2X_{12}$ (M = Ge, Si, Sn, Al or P, and X = O, S or Se) family of superionic conductors. *Energy Environ. Sci.* **6**, 148–156 (2013).

[26] Kato, A. *et al.* Mechanical properties of sulfide glasses in all-solid-state batteries. *J. Ceram. Soc. Jpn.* **126**, 719–727 (2018).

[27] Rabadanov, M. K., Pietraszko, A., Kireev, V., Ivanov-Schitz, A. & Simonov, V. Atomic structure and mechanism of ionic conductivity of Li 3.31 Ge 0.31 P 0.69 O 4 single crystals. *Crystallogr. Rep.* **48**, 744–749 (2003).

[28] Kamaya, N. *et al.* A lithium superionic conductor. *Nat. Mater.* **10**, 682 (2011).

[29] Materials cloud archive. `https://archive.materialscloud.org/record/2021.15`.
